# Perceptual Learning of Noise-Vocoded Speech Under Divided Attention

**DOI:** 10.1177/23312165231192297

**Published:** 2023-08-07

**Authors:** Han Wang, Rongru Chen, Yu Yan, Carolyn McGettigan, Stuart Rosen, Patti Adank

**Affiliations:** 1 Department of Speech, Hearing and Phonetic Sciences, 4919 University College London, London, UK

**Keywords:** perceptual learning, noise-vocoded speech, divided attention, task difficulty, phonological processing, lexical processing

## Abstract

Speech perception performance for degraded speech can improve with practice or exposure. Such perceptual learning is thought to be reliant on attention and theoretical accounts like the predictive coding framework suggest a key role for attention in supporting learning. However, it is unclear whether speech perceptual learning requires undivided attention. We evaluated the role of divided attention in speech perceptual learning in two online experiments (*N* = 336). Experiment 1 tested the reliance of perceptual learning on undivided attention. Participants completed a speech recognition task where they repeated forty noise-vocoded sentences in a between-group design. Participants performed the speech task alone or concurrently with a domain-general visual task (dual task) at one of three difficulty levels. We observed perceptual learning under divided attention for all four groups, moderated by dual-task difficulty. Listeners in easy and intermediate visual conditions improved as much as the single-task group. Those who completed the most challenging visual task showed faster learning and achieved similar ending performance compared to the single-task group. Experiment 2 tested whether learning relies on domain-specific or domain-general processes. Participants completed a single speech task or performed this task together with a dual task aiming to recruit domain-specific (lexical or phonological), or domain-general (visual) processes. All secondary task conditions produced patterns and amount of learning comparable to the single speech task. Our results demonstrate that the impact of divided attention on perceptual learning is not strictly dependent on domain-general or domain-specific processes and speech perceptual learning persists under divided attention.

## Introduction

Perceptual learning is the improvement in task performance resulting from exposure to sensory input (Goldstone, 1998). Such improvement is often observed for speech recognition in suboptimal listening situations. Listeners in these situations may encounter talkers with an unfamiliar accent (e.g., Banks et al., 2015b), fast talkers (Dupoux & Green, 1997), background noise (e.g., Song et al., 2012), or a competing talker (e.g., Mesgarani & Chang, 2012). Despite the degradation of speech input, speech perception can improve with short exposures to a novel degradation as characterised by faster response times (RTs) and higher accuracy.

This improvement in perception is observable with mere exposure to the speech signal for a wide range of unfamiliar and/or degraded speech signals: time-compressed speech (Dupoux & Green, 1997; Fairbanks & Kodman, 1957), accented speech (Adank & Devlin, 2010; Banks et al., 2015b), speech embedded in noise (Cainer et al., 2008; Song et al., 2012), and noise-vocoded speech (Davis et al., 2005; Hervais-Adelman et al., 2008; Kennedy-Higgins et al., 2020; Paulus et al., 2020). For example, listeners can generally achieve a significant improvement (∼ 30% increase in accuracy, or faster RT) for these degradations, even within five trials of exposure to novel stimuli (Cooke et al., 2022). In contrast, perceptual learning can also occur over a long period (e.g., hundreds of sentences over multiple sessions), in a variety of challenging listening conditions, such as time-compressed speech (Banai & Lavner, 2012), accented speech (Weber et al., 2014), noise-vocoded speech (Rosen et al., 1999), and speech in noise (Green et al., 2019). The effect of learning can persist even relatively long after the training ends (e.g., sustained improvement in speech-in-noise perception four weeks after post training; Green et al., 2019), signalling a long-term plastic change in perception (Kumano & Uka, 2013). In the current study, we focus on the former, the rapid type of perceptual learning that happens in a short exposure window.

Studies on speech perceptual learning used noise-vocoding to evaluate how listeners adapt to the degradation of the speech signal (Shannon et al., 1995). A noise-vocoder uses the amplitude envelopes extracted from separate frequency bands (typically between 1 and 32; McGettigan et al., 2014) of the speech signal to modulate the corresponding bands of a carrier signal (e.g., white noise). This procedure, therefore, removes spectral detail while preserving low-frequency amplitude and temporal information. This type of degradation has been used to simulate the speech processing of cochlear implant users (Faulkner et al., 2000; Rosen et al., 1999). In normal-hearing listeners, the intelligibility of the signal increases logarithmically with the number of bands, meaning that performance over bands increases more for lower numbers of bands (Shannon et al., 2004). In a typical paradigm, listeners are presented with vocoded sentences and any improvement in recognition performance with additional exposure indicates learning. For degradation of six bands, an improvement of 10%–15% in word recognition performance over 20 sentences has been reported (Huyck et al., 2017; Wayne & Johnsrude, 2012), whereas other studies showed more robust learning, for example, a 40% increase in correctly reported words over 30 sentences (Davis et al., 2005).

The cognitive mechanisms supporting the learning process remain poorly understood. Specifically, it is unclear to what extent perceptual learning is dependent on attention. Attention is defined as a cognitive function with limited capacity that selects and controls incoming information (Pashler, 1998, p. 3), and is involved in processing input from different sensory domains (e.g., vision and audition; Scarf, 1998). It has been proposed that perceptual learning of speech relies on attentional processes (Goldstone, 1998; Huyck & Johnsrude, 2012). Hunter and Pisoni (2018) demonstrated that the role of undivided attention in perceiving noise-vocoded speech can be established using a dual-task paradigm. They found that word report was less accurate for a high-load than for a low-load domain-general dual task (i.e., recall of seven vs. three onscreen digits). Therefore, attention can be divided systematically by keeping the difficulty of the primary (speech) task constant while varying the secondary task's difficulty (Gennari et al., 2018; Navon & Gopher, 1980). Because speech performance deteriorated under a hard (as opposed to an easy) dual task, the resources occupied by the secondary task must also be competed for by recognising noise-vocoded speech (Kahneman, 1973). Hunter and Pisoni further found that the effect of task load on word report depends on acoustic degradation and word predictability—divided attention substantially deteriorated word recognition for four bands with high predictability, and eight bands with low predictability, but had little effect on the opposite situations. Although there may have been ceiling and floor effects that obscured the load effects in these opposite situations, there was limited evidence to support this explanation in the data as argued by the authors. Therefore, these results indicate that attention selectively facilitates processing at the level where robust evidence is available for making inferences on the sensory inputs (i.e., acoustic details for eight-band speech with low predictability and lexical information for four-band speech with high predictability).

Huyck and Johnsrude (2012) showed that attention not only modulates the perception of degraded speech, but also the perceptual learning of degraded speech. In a between-group training session, participants selectively attended to noise-vocoded sentences and repeated back the words they heard, or selectively attended to concurrent auditory bursts or visual ellipses and decided whether a target pattern was presented. Before and after training, all participants conducted a testing phase where they completed the speech task. The authors found that adaption to noise-vocoded speech only occurred when attention was selectively directed to the speech task, rather than the concurring auditory and visual distractors. Huyck and Johnsrude’s (2012) results suggested an essential role of attention in perceptual learning of speech.

Hervais-Adelman et al. (2008) established the level of processing where perceptual learning of noise-vocoded speech occurs and concluded that learning occurs at a stage where physical features are abstracted to higher-level representations. They tested listeners’ recognition of noise-vocoded words before and after training the listeners with a separate set of noise-vocoded words or nonwords. Listeners trained with words and nonwords improved the same over the two test sessions and therefore the results supported a sub-lexical locus for learning. A follow-up study by Hervais-Adelman et al. (2011) further illustrated that exposure to low-pass noise-vocoded speech improved the perception of subsequent high-pass speech. Critically, Mattys et al. (2014) showed that listeners’ capacity to discriminate a phoneme in noise decreased linearly with the increase of visual distractors in a concurrent visual search task. These results suggest that perceptual learning of noise-vocoded speech and the effects of a secondary visual task on speech perception result in changes to acoustic-phonetic processing of the degraded input. These results imply that the processing load posed by a secondary task can affect the low-level sensory processing that is also needed for the acoustic processing of speech.

Besides the empirical findings discussed above, several theoretical frameworks, including Goldstone's framework for perceptual learning (Goldstone, 1998), Amitay's reverse hierarchy theory (RHT; Amitay, 2009), and Friston's predictive coding theory (Feldman & Friston, 2010), have also formalised the relationship between attention and perceptual learning. These frameworks converge on the idea that attention modulates perception through a top-down process by elevating the salience of a fraction of low-level sensory cues over time. Goldstone (1998) and Amitay (2009) proposed that perceptual learning may result from shifting attention from task-irrelevant cues to task-relevant cues, leading to easier access to low-level representations. In comparison, Feldman and Friston (2010) presumed that the computational operation of attention on learning is subject to the integration of top-down predictions and bottom-up sensory input. The balance between the weights of the two streams of information is overseen by the reliability of the prediction error. When the sensory input is reliable (e.g., 16-band speech), prediction errors would result in updating the model. However, when the input is less reliable (e.g., four-band speech), the perceptual system is more likely to rely on its previous experience (e.g., lexical knowledge) to guide perception. For example, the written text of the speech content presented before a noise-vocoded word enhances intelligibility, but the effect is much larger (i.e., 80% greater enhancement in self-reported speech clarity) for moderately degraded (i.e., four bands), compared to mildly degraded speech (i.e., eight bands; Sohoglu et al., 2014). Hypothetically, attention selectively samples the highly reliable prediction errors, which then become more influential in refining the model (Feldman & Friston, 2010; Lupyan & Clark, 2015). Thereby, the variance in the prediction reduces and the proximity of the prediction to the sensory input over time is manifested as perceptual learning (Friston, 2009; Sohoglu & Davis, 2016). Thus, attention supports perceptual learning of speech by minimising prediction errors.

Two issues are unresolved regarding the relationship between attention and speech-perceptual learning. First, it is unclear whether perceptual learning of degraded speech relies on undivided attention or whether it can also occur under divided attention, for example, in the presence of a dual task. Huyck and Johnsrude's results are restricted to situations where attention is entirely directed to the speech signal or entirely exhausted by other tasks. For instance, it is unclear whether participants in Huyck and Johnsrude showed no perceptual learning because they selectively attended to distractors, or because they did not perform the speech task. Therefore, it is unknown if and how attention interacts with perceptual learning in speech processing upon encountering both signal degradation and the processing load posed by a dual task. Second, it is unclear whether attention supports perceptual learning of speech in a domain-general or domain-specific manner. Studies conducted thus far have all used a domain-general secondary task (e.g., visual search, digit recall) to load on speech processing. It is therefore unclear whether the linguistic level of processing (e.g., lexical or phonological) impacts on perceptual learning of speech. The theories outlined above do not make specific predictions regarding either issue, as the nature of attentional processing in perceptual learning is underspecified in all of them. The existing theories do not postulate whether learning can occur under divided attention and neither do they make predictions regarding the domain-specificity of the attentional processes engaged.

### The Current Study

The current study aimed to establish whether perceptual learning of speech can occur under divided attention and whether the attentional processes involved are domain-general or domain-specific in nature. Experiment 1 examined whether and how the difficulty level of a domain-general secondary task affected perceptual learning. Participants were divided into four groups: a baseline group heard and repeated back noise-vocoded speech (single task). Participants in three other groups performed the same speech task under divided attention (dual task): while conducting a concurrent domain-general (visual) task at three difficulty levels (easy, intermediate, or hard). We tested three hypotheses related to the relationship between divided attention and perceptual learning of speech. Hypothesis 1 predicted that divided attention eliminates perceptual learning. Hypothesis 2 predicted that dividing attention modulates the perceptual learning process parametrically depending on the difficulty level of the dual task. Hypothesis 3 predicted that divided attention does not affect perceptual learning.

Experiment 2 aimed to clarify the domain-specific nature of attentional resources required for perceptual learning. Three groups of participants conducted the speech task from Experiment 1 while completing a dual task that recruited phonological, lexical, or visual processes. We predicted that domain-specific (phonological or lexical) processes would have a larger impact than domain-general (visual) processes in the perceptual learning of speech, as listeners are expected to rely heavily on lexical processing when speech input is moderately degraded (Sohoglu & Davis, 2016; Sohoglu et al., 2014).

## Experiment 1

### Experiment 1: Methods

#### Participants

One-hundred and ninety-two participants (160 females [F] and 32 males [M] between 18 and 35 years of age [Y], mean = 26.2Y, standard deviation [*SD*] = 5.1Y) completed Experiment 1. All self-declared to be monolingual British English speakers residing in the United Kingdom at the time of the experiments. All reported normal hearing and no neurological disorders (including dyslexia). Participants were assigned to one of four conditions (*n* = 48 per condition). The demographics for each condition were: single-task condition (40F, mean = 25.5Y, *SD* = 5.3Y), dual easy condition (40F, mean = 27.5Y, *SD* = 4.9Y), dual intermediate condition (40F, mean = 25.5Y, *SD* = 5.3Y), dual hard condition (40F, mean = 26.2Y, *SD* = 4.6Y). The sample size per condition and the sex ratio (5F:1M) were based on our recent in-lab (Banks et al., 2021) and online (Trotter et al., 2021) studies that investigated perceptual learning of noise-vocoded speech in a between-group design. After collecting an initial 192 participants, we conducted a post-experiment screening and recruited new participants to replace: (1) participants whose performance (see the ‘Dependent Measures’ section) in the speech or visual task were three SDs away from the group mean; (2) those whose response accuracy in the visual task was below chance level (i.e., < 50% correct, 32 participants); (3) seven participants who conducted the experiment in a noisy environment as judged from the recorded speech task responses. All participants were recruited via the online recruitment platform Prolific (Peer et al., 2017) and paid at a rate corresponding to £7.50 per hour. The experiment was approved by the Research Ethics Committee of University College London (#0599.001).

#### Materials

##### Primary Task

The primary, speech recognition, task used sentences from the Bamford Kowal-Bench (BKB) corpus (Bench et al., 1979) produced by a female speaker. The recordings were collected in an anechoic chamber at UCL using a Type 4190 microphone on a Brüel & Kjær 2231 Sound Level Meter (sampling at 16 bit and 22.05 kHz), which was connected to a Sony 60ES digital audio tape recorder.

The BKB corpus consists of 336 sentences, each with three to four key words. Forty-two sentences containing three key words were drawn from the original set of 336. Each key word was unique across the 42 sentences, and words that only differed in the suffix (e.g., oven vs. ovens) or had minor morphological deviants (e.g., they vs. they’re) were counted as duplicates. The sentence set was first normalised to the same root-mean-square amplitude (70 dB; Kennedy-Higgins et al., 2020) in Praat (version 6.1.42; Boersma, 2001) before being processed by a noise vocoder adapted from Rosen et al. (1999) in MATLAB (version R2021a; MathWorks). Forty sentences (for the main trials, also see the ‘Procedure’ section) were band-pass filtered into six logarithmically spaced frequency bands between 50 and 5,000 Hz following Greenwood (1990)'s frequency-position function. The frequencies of the lower band edges were 50, 200, 456, 889, 1,626, and 2,876 Hz. Two sentences (for the familiarisation trials) were filtered into 15 bands (between 50 and 5000 Hz). Each band's amplitude envelope was extracted using a low-pass filter (cut-off at 300 Hz) followed by rectification. This envelope was used to modulate a white noise, which was then filtered by the same band-pass filter used to extract the envelope, before all the band outputs were summed together.

##### Secondary Task

**Figure 1.**
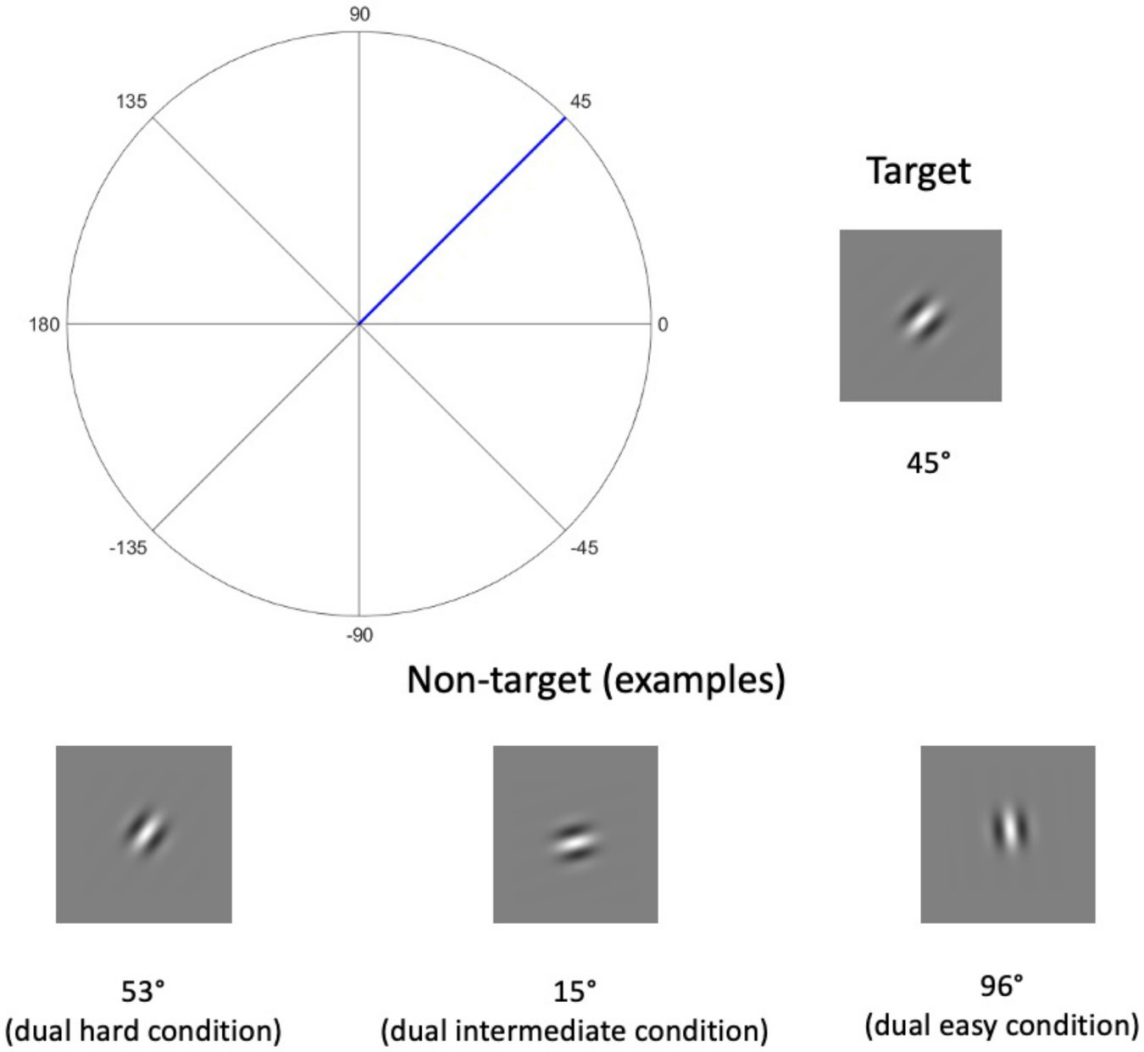
The orientation of a Gabor patch is defined as the angle formed by the horizontal axis and the patch. The target orientation (45° clockwise from vertical) is highlighted as the blue line on the coordinates. The patches are presented through Gaussian windows so that the light intensity decreases (in a Gaussian manner) at the edges of a patch. The plots of patches are for illustration purposes and thus not scaled to their actual size. These examples do not exhaust all possible orientations of a non-target patch in Experiment 1.

The secondary task was a visual decision task where participants judged the orientation of a Gabor patch (Calder-Travis & Ma, 2020). Each patch was a sine wave grating presented through a Gaussian window with an SD of 0.16 cm and a frequency of 2.80 cycles per cm (Figure 1). All stimuli were displayed on a grey background (RGB = [128, 128, 128]). Peaks and troughs of the sine waves took the possible maximum and minimum RGB values ([255, 255, 255] and [0, 0, 0], respectively) at the centre of the Gaussian window. We also adjusted the phase of these Gabor patches to ensure there was always a peak of the sine wave at the centre of the Gaussian window.

Each Gabor patch was located on the circumference of an imaginary circle that had a radius of 3.16 cm. The centre of the circle is aligned with the centre of the monitor. The location of a Gabor patch on the circle was randomly drawn from a uniform probability distribution so that a patch was equally likely to be anywhere on the circle. The stimuli were produced with a customised MATLAB script.

### Procedure

The experiment was hosted on Gorilla.sc, an online testing environment (Anwyl-Irvine et al., 2020). Participants were first asked to report their monitor size before a customised JavaScript detected their display resolution. One-hundred and two participants who reported a monitor size smaller than 10.1 inches in diagonal or had a resolution less than 1024*768 were disqualified from participation to ensure the stimuli could be displayed in the desired size without truncation across all participants.

Those who passed the display validation were provided further information about the experiment and asked to provide consent. They were requested to turn on the auto-play of audio and video and enable cookies for Gorilla.sc. Participants were required to plug in their headphones and not use wireless (Bluetooth) headphones. To exclude those who were not wearing headphones, participants passed a headphone screening (Woods et al., 2017). Then, they were presented with 1000 ms of white noise, which they were allowed to replay to adjust their volume to a comfortable level. The final validation was a microphone check where participants were asked to record their own voices to check if their responses could be recorded.

Before the main experiment phase, a customised JavaScript enabled full-screen mode and hid all window components of the browser (i.e., the tabs, address bar, and bookmark bar). Then, a tool provided by Gorilla.sc guided participants to calibrate their monitor so that the Gabor patches could be displayed at an equivalent size (also see the ‘Materials’ section) across all participants regardless of their monitor size and resolution. Participants were asked to place a standard-size credit card against an image of the card shown on the monitor and to drag a slider until the size of the image matched that of the physical card. The calibration programme then used the pixel (px) counts of the image to acquire the px density (px per inch) of the monitor and scaled the Gabor stimuli to the width (in px) corresponding to the desired size (in cm). Participants were then presented with a 45° Gabor patch (Figure 1) which illustrates the target orientation for the stimuli of the secondary task.

**Figure 2.**
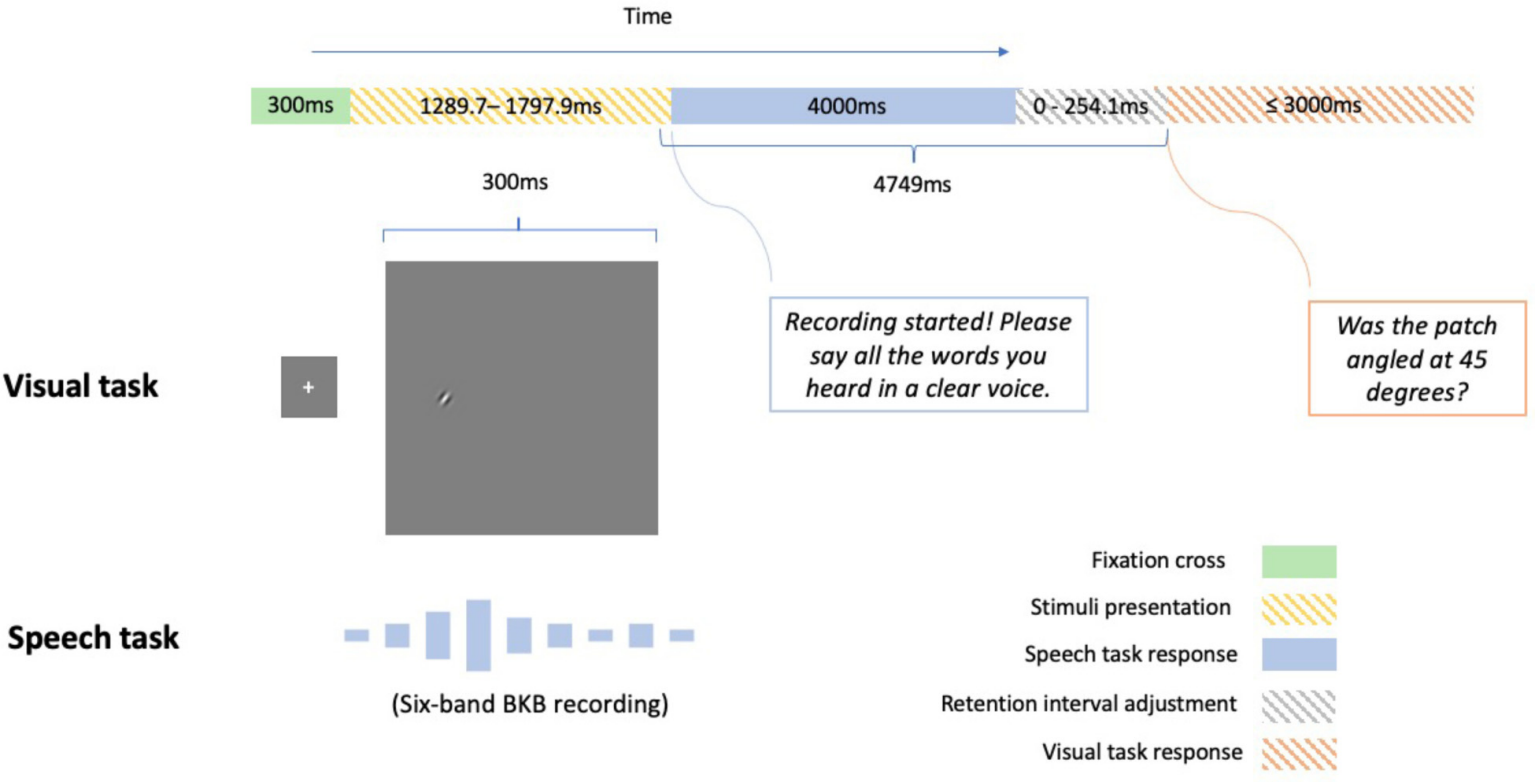
In Experiment 1, participants performing the dual-task heard a six-band Bamford Kowal-Bench (BKB) sentence while they judged the orientation of a Gabor patch presented briefly. They then had to verbally report the words they heard and whether the Gabor patch was oriented at 45° clockwise from vertical. A retention interval adjustment was added between the responses to the speech task and the visual task so that the duration between the end of the Gabor patch presentation and the start of the visual-task response was identical across trials. The plots of the fixation cross and the Gabor patch are for illustration only and not scaled to their actual size. The exemplar patch does not exhaust the possible locations (see the ‘Materials’ section) of a patch in the experiment.

In the main experiment, participants performed two familiarisation trials before the 40 experimental trials. The procedure for the familiarisation and the main trials was identical, except the familiarisation stimuli were highly intelligible (i.e., 15 bands) and the correct answer was revealed after the participant spoke their response. After the two 15-band familiarisation trials, participants heard one 6-band BKB sentence ‘The two farmers are talking’ spoken in Maltese (‘Iż-żewġ bdiewa qed jitkellmu’) by a female speaker to familiarise the participants with the acoustic degradation of the main trials. The procedure for a main sentence trial in the dual task is illustrated in Figure 2.

Participants in all dual-task conditions repeated a noise-vocoded sentence while judging whether a Gabor patch was angled at a target orientation (45° clockwise, Figure 1). Participants were not instructed to prioritise either of the two tasks and were only told to perform both tasks together, as it would be hard to prevent participants from dynamically changing their allocations of resources over time, which might be particularly true for a real-life scenario. In each trial, a fixation cross was displayed at the screen's centre for 300 ms. They then heard a six-band BKB sentence plus a Gabor patch presented for 300 ms. The Gabor patch appeared 150 ms prior to the midpoint of the sentence duration and ended at 150 ms following the midpoint. Subsequently, participants were given 4 s to repeat back the sentence. Afterwards, participants were prompted to report whether the Gabor patch displayed the target orientation. Participants had 3 s to respond by pressing the left (‘target present’) or right (‘target absent’) arrow keys. Because sentences were of different durations, a blank window of variable duration (0–254 ms) was interleaved between the spoken and the key press responses to ensure each trial had the same retention interval (4749 ms) between the end of the Gabor patch presentation and the start of the visual-task response. Participants in the single-task condition only heard and responded to the speech stimuli, and each trial terminated after the spoken response window.

In the secondary task, target orientation (45°) was present in 50% of trials. The task difficulty was manipulated by varying the difference in orientation between the target and non-target trials. The ranges of difference in orientations between a non-target and a target (Δ) in each condition were as follows (Figure 1): 48° < Δ ≤ 60° (dual easy condition), 24° < Δ ≤ 36° (dual intermediate condition) and 0° < Δ ≤ 12° (dual hard condition). The orientations of the non-target Gabor patches came from a uniform distribution so that all possible non-target orientations were equally likely to enter the sample. The location of a Gabor patch varied from trial to trial to prevent participants from using a tool (e.g., a ruler or sticker) to help decide orientations. For each participant, the trial order was randomised, yet the pairing between a sentence and a Gabor patch in a trial was the same.

After the main experiment, participants completed a questionnaire where they indicated how much effort and attention they invested on a 0 to 100 scale (see Supplemental Appendices C to E for details and analyses). The experiment took 23 min [*SD* = 13.3 min] on average.

### Dependent Measures

The percentage of correctly recognised key words for each sentence was the main dependent measure. Following Banks et al. (2021), words with incorrect suffixes (e.g., -s, -ed, -ing) were scored as correct, but words (including compound words) reported in part (e.g., ‘raindrops’ instead of ‘raincoats’) were scored as incorrect. Trials without a response were coded as 0% correct. RTs in milliseconds and correctness of response (i.e., 0 or 1) on each trial were measured for the visual task to describe the change in performance over the whole task. Trials having an incorrect or no response for the dual task were excluded from the RT analysis. Finally, subjective ratings were collected from the questionnaire to describe the effort and attention that participants invested in each of the dual tasks.

### Analysis

We fit a set of generalised linear mixed-effect models (GLMMs) using the glmer() function in the lme4 R-package (version 1.1–27.1; Bates et al., 2015) to uncover the relationship between the predictors and the behavioural responses in the main experiment. We analysed the % correct data from the sentence recognition task, and the correctness and RTs for the visual tasks. RT models assumed a gamma distribution of residuals and used a log-link function to account for the skewed RT distributions (Lo & Andrews, 2015), while models for % correct and correctness assumed binomially distributed residuals and adopted a logit link function.

All models had Task (i.e., single, dual easy, dual intermediate, and dual hard for speech % correct, and dual-easy, dual-intermediate, dual-hard for visual-task correctness and RTs), Trial, and their interaction as predictors. The performance and speed improvement over trials (except for visual-task accuracy) seemed to be greater at early than at later trials, which was in line with Cooke et al. (2022), a study that found a logarithmic trend of rapid perceptual learning across a wide variety of degraded speech. For visual-task accuracy, a quadratic function was likely to fit the data as there seemed to be a drop in performance in late trials after the performance reached its peak. Akaike information criterion (AIC), a measure for goodness-of-fit (i.e., maximised log-likelihood) while penalising for a complex model (Burnham & Anderson, 2004), further confirmed that applying a transformation to Trial (logarithmic or quadratic) yielded a significantly smaller AIC (i.e., a better fit) compared to a model without transformation (see Supplemental Table B1 for model comparison details). Therefore, a logarithmic transformation ( $\log_{e}\;x$ ) was applied to Trial in all but the visual accuracy model, and a second-degree polynomial term for Trial ( $x^{2}$ ) was added on top of its linear fit to the visual accuracy model.

The model for speech % correct initially included random intercepts for Participant and Sentence, and random slopes for Trial by Participant and Task by Sentence. The maximal models for visual-task correctness and RTs included random intercepts for Participant and Gabor Prompt, and random slopes for Trial by Participant and Task by Gabor Prompt. To select an optimal fitting model for our data, we first removed random effects that caused a convergence failure (Mickan et al., 2020). Next, we excluded the random effects whose inclusion yielded inaccurate estimates of the raw responses – a sign of over-fitting (Nannen, 2003). Lastly, we applied a backward model-selection procedure using the anova() function, which compared the goodness-of-fit (i.e., maximised log-likelihood) of two models given the data while penalising for the complexity of the models. Each time we performed a comparison between a model and a simpler model excluding a certain random effect and removing the effect from the model where it did not significantly contribute to the model fit. We continued such comparisons until we found the best-fitting model. The best fitting model for speech % correct included random intercepts for the Participant and random slopes for Trial by Participant. The final model for visual-task correctness included random intercepts for Prompt and random slopes for Trial by Participant. The model for visual RT had random intercepts for Participant and Prompt.

### Experiment 1: Results

#### Speech Task

**Figure 3.**
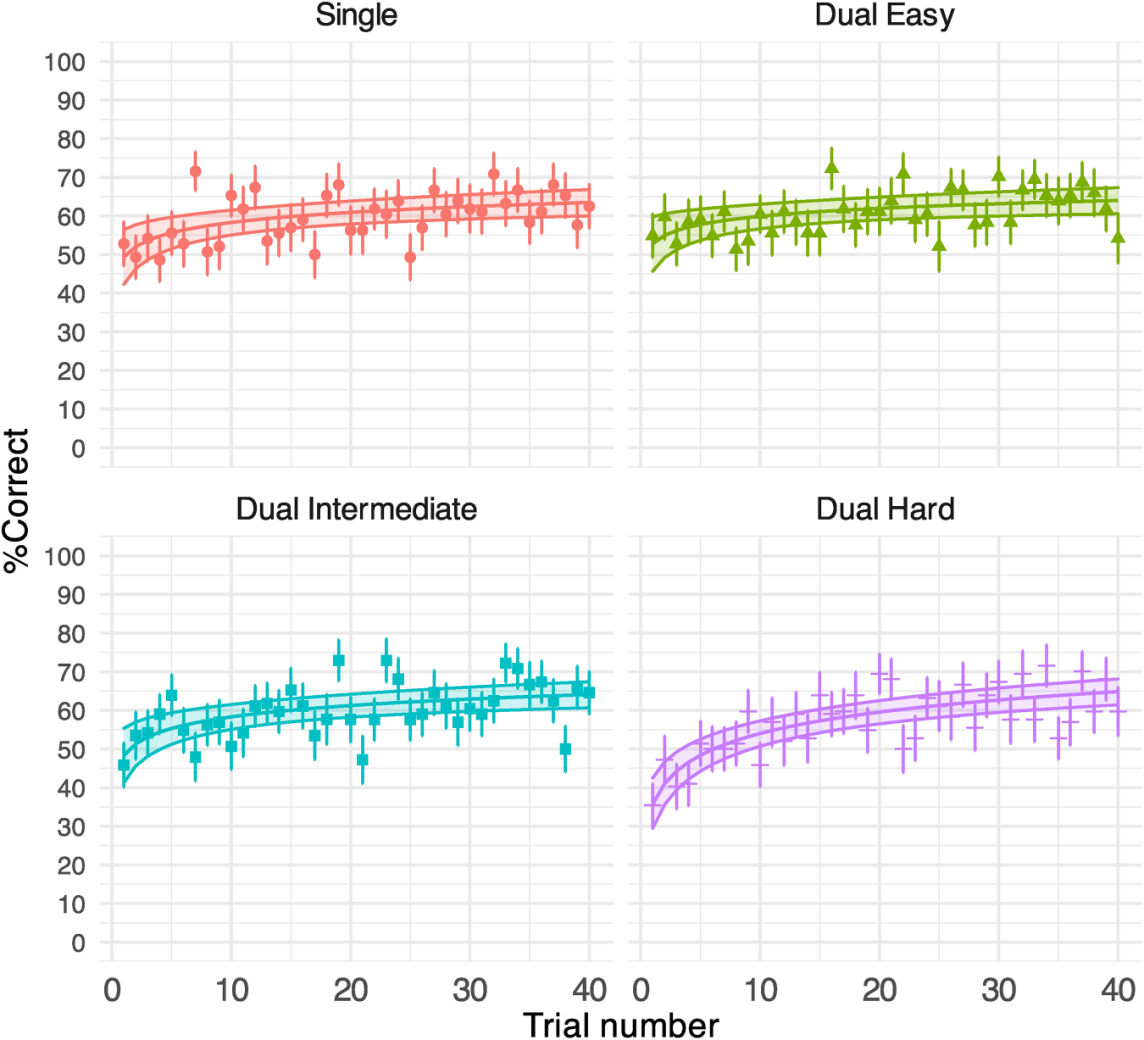
Generalised linear mixed-effect model (GLMM)-estimated percent of correctly reported key words in Experiment 1 displayed as a function of the trial (middle solid lines in the coloured areas). Each panel illustrates the results under each task condition. Filled areas represent 95% confidence intervals. Points denote the raw mean % correct obtained on each trial. Error bars indicate the standard error of the mean.

Table 1 shows the GLMM outputs. Figure 3 illustrates the % correct of key words reported by Trial by Task and the predictions of the model. Visual task difficulty significantly modulated speech task accuracy – the overall sentence recognition performance under the hard visual task (57.36%, *SD* = 10.00) was significantly lower than that in other conditions (dual easy: 60.80% [*SD* = 9.21]; dual intermediate: 59.88% [*SD* = 9.84]); single task: 59.57% [*SD* = 11.71], Table 1). All four groups showed a significant effect of Trial on performance (all *p*'s < .01). The fitted functions in Figure 3 further confirmed that this effect reflects an improvement of performance over time. Therefore, all four groups showed significant perceptual learning of speech in 40 trials.

**Table 1.** Model Outputs for the GLMM Assessing the Fixed Effects of Task and Trial on the Speech Task Accuracy in Experiment 1.

Fixed effects				
	*β*	*SE*	*z*	*p*
(Intercept)	−0.59	0.15	−4.04	**<**.**001**
log(trial)	0.33	0.05	7.00	**<**.**001**
speech_single [dual_hard]	0.56	0.21	2.74	.**006**
dual_intermediate [dual_hard]	0.52	0.21	2.53	.**011**
dual_easy [dual_hard]	0.70	0.21	3.40	**<**.**001**
log(trial):speech_single [dual_hard]	−0.17	0.07	−2.57	.**010**
log(trial):dual_intermediate [dual_hard]	−0.15	0.07	−2.28	.**022**
log(trial):dual_easy [dual_hard]	−0.20	0.07	−3.05	.**002**

Abbreviations: GLMM = generalised linear mixed-effect model; *SE* = standard error.

The reference level is shown in a bracket. *P* values less than 0.05 are marked in bold.

We established if and how the pattern of perceptual learning was subject to the presence and difficulty of a secondary task by examining the interaction between Task and Trial. The speech task under the hard visual condition had a larger performance gain per trial than all other conditions (Table 1), which can be seen by the steeper improvement in early trials under the hard condition. The difference in trends of learning across conditions also contributed to the magnitude of learning. According to the fitted functions, listeners under all conditions except dual hard showed a comparable increase in performance: single (14.11%), dual easy (7.53%) and dual intermediate (12.05%). The perceptual learning of speech was robust under the most challenging condition (29.14%), as demonstrated by a lower performance and more learning in early trials. By the end of the task, listeners in all groups achieved ∼ 65% correct.

#### Visual Task

**Figure 4.**
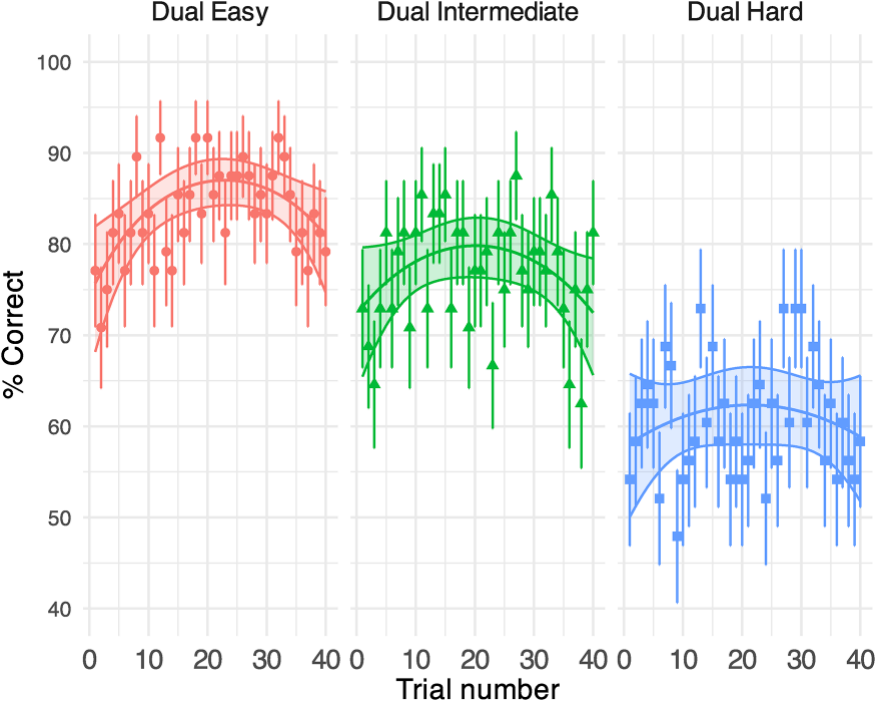
Generalised linear mixed-effect model (GLMM)-estimated percent of correct responses for the visual task at different task difficulty in Experiment 1, displayed as a function of the trial (middle solid lines in the coloured areas). Each panel illustrates the results under each task condition. Filled areas represent 95% confidence intervals. Points denote the raw % correct of response (i.e., number of correctly responded participants/total number of participants * 100) on each trial. Error bars indicate the standard error of the % correct.

**Figure 5.**
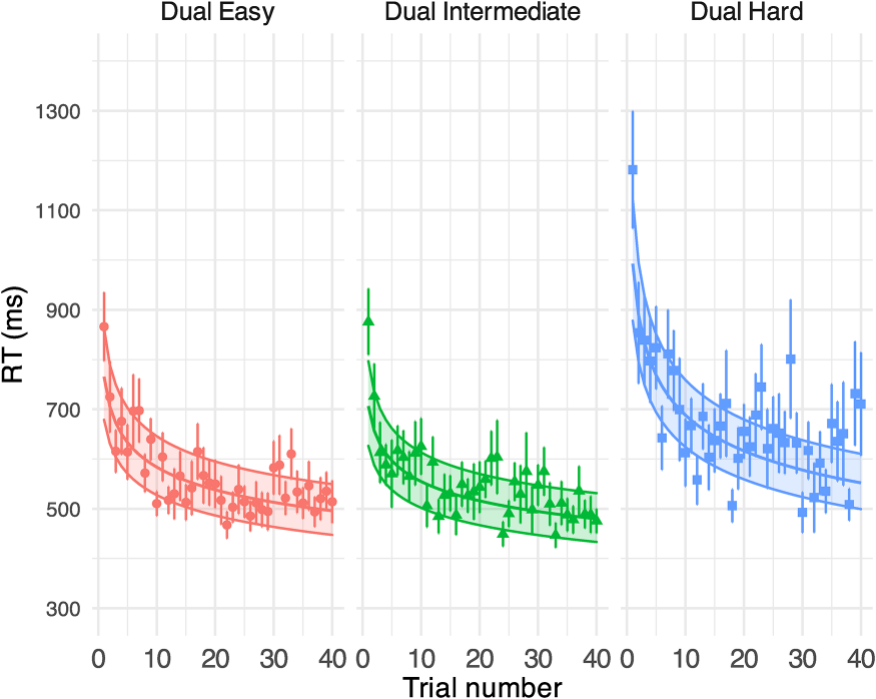
GLMM-estimated visual task RTs in a millisecond at different task difficulties in Experiment 1, displayed as a function of the trial (middle solid lines in the coloured areas). Each panel illustrates the results under each task condition. Filled areas represent 95% confidence intervals. Points denote the raw mean RTs for correct visual task responses obtained on each trial. Error bars indicate the standard error of the mean. See Supplemental Table B3 for model output.

Abbreviations: GLMM = generalised linear mixed-effect model; RTs = response times.

The GLMM model on the response correctness (displayed in % correct) in the visual tasks (Figure 4) revealed a robust effect of task difficulty (see Supplemental Table B2 for model outputs): participants conducting a moderately difficult visual task outperformed those who received the hardest task but had a significantly lower performance than those in the easiest condition. Importantly, the average performance in all groups (dual easy: 83%, dual intermediate: 77%, dual hard: 61%) was above the chance level (0.5), signalling the allocation of attentional resources on visual tasks. The trial did not modulate the visual performance in the most difficult condition but had a quadratic impact on the easiest and moderately difficult conditions [dual easy: *β* (*SE*)* =* −14.376 (4.910), *p* = .003; dual intermediate: *β* (*SE*)* =* −9.282 (4.578), *p* = .043], where accuracy increased over the first 20 trials but declined thereafter. See Figure 5 for the RT results.

### Experiment 1: Discussion

We divided participants’ attention to sentence recognition parametrically using a domain-general visual dual-task with three difficulty levels. Despite having fewer attentional resources available for speech perception in harder visual conditions (see the self-reported attention rating in Supplemental Figure C4), sentence recognition performance improved as much or more as in the isolated control task (Figure 3), meaning perceptual learning of noise-vocoded speech can occur under divided attention. However, the pattern of perceptual learning was different under the most challenging visual condition: the improvement was larger in this condition and was associated with more rapid learning at the beginning of the experiment.

Our data did not support Hypothesis 1 – divided attention stops perceptual learning, as an adaptation to speech was observed under all task conditions. The results partially supported Hypothesis 2 – dividing attention modulates the perceptual learning process parametrically – as the gradient of learning depended on secondary task difficulty. However, contrary to our expectations, learning was greater, not diminished, with the more difficult task. Hypothesis 3 – divided attention does not affect perceptual learning – was not supported, as speech adaptation had a different pattern of performance change over trials under the hard visual task. Therefore, our results extended existing theories and findings by showing that selective attention is not required for speech-perceptual learning.

Experiment 1 used a domain-general task, and it remains unclear whether speech perceptual learning persists when attention is diverted by a secondary task requiring domain-specific processes also used in speech recognition, that is, phonological or lexical processes. Experiment 2 examined whether perceptual learning of noise-vocoded speech depends on domain-specific or domain-general processing. In Experiment 2, groups of participants completed the speech task from Experiment 1 while performing a dual task designed to engage domain-specific (phonological, lexical), or domain-general (visual) attentional processes. Based on assumptions from the predictive coding framework, we used the same set of highly predictable sentences from Experiment 1 in the speech task to maximise the potential lexical benefits and expected that participants would rely on lexical information over acoustic, spectral details for sentence recognition (McGettigan et al., 2014). The phonological secondary task was a syllable-counting task as the sub-lexical, syllable-level processing was shown to improve the adaption to noise-vocoded speech (Hervais-Adelman et al., 2008). The lexical task was a semantic decision task as the processing of word-level meaning engaged in this task seems to also benefit the learning of noise-vocoded speech (Davis et al., 2005).

Hypothesis 1 predicted that perceptual learning is not dependent on domain-general nor domain-specific resources. Hypothesis 1 is supported if perceptual learning of speech is similar under all three dual-task and single-task conditions. Hypothesis 2 predicted that learning of speech relies critically on language processes in general. If Hypothesis 2 is supported, learning should be impaired equally more under both language tasks than under the visual task. Hypothesis 3 predicted that lexical processing is more influential than phonological processing in learning these highly predictable and moderately degraded sentences (Feldman & Friston, 2010; Sohoglu & Davis, 2016; Sohoglu et al., 2014). Support for Hypothesis 3 would be shown by impaired perceptual learning under the lexical task compared to under both the visual and phonological tasks.

## Experiment 2

### Experiment 2: Methods

#### Participants

One-hundred and forty-four participants (120F and 24M, 18–35Y, mean = 24.3Y, *SD* = 4.5) who did not take part in Experiment 1 were included in Experiment 2. We replaced participants whose performance in the speech or secondary task was three SDs away from the group mean (six participants), those whose response accuracy in the secondary task was below chance level (five), those who conducted the experiment in a noisy environment (six), and those who participated in Experiment 1 (two). Participants were randomly assigned to the following groups of 48 with a same-sex ratio (5F:1M) as that in Experiment 1: visual condition (mean = 25.2Y, *SD* = 4.8Y), phonological condition (mean = 23.6Y, *SD* = 4.6Y), lexical condition (mean = 24.1Y, *SD* = 4.1Y). The recruitment platform and participants’ demographic profiles were as in Experiment 1. We used the data collected for the single speech task in Experiment 1 as the baseline speech performance for Experiment 2. Therefore, no additional participants were recruited for the single-task condition.

#### Materials

##### Primary Task

The same set of 42 noise-vocoded BKB sentences (40 with six bands, two with 15 bands) from Experiment 1 were used for the primary (speech) task in Experiment 2.

##### Secondary Task

Stimuli for the secondary tasks were a set of words taken from SUBTLEX-UK, a word frequency database of British English based on television subtitles (van Heuven et al., 2014). We extracted nouns of medium to high-frequency use (which have a Zipf measure of 3–4.5; see van Heuven et al., 2014) and further selected two-syllable and three-syllable words with the meaning of either animal or man-made object (e.g., leopard, kangaroo, boiler and camera). The final stimulus set contained 42 words – 40 for main trials, and two for familiarisation. The 40 main-trial words (see Supplemental Table A1) were balanced for their syllable counts and semantic category. That is, we had 10 words for each of these four subsets: two-syllable animal, two-syllable objects, three-syllable animal, and three-syllable objects.

The stimuli were presented as visual words on the monitor. The stimulus in each trial was a black word (height = 0.65 cm) displayed on a white background. Mimicking the dual intermediate condition of Experiment 1, we further manipulated the orientation of these words – half were 45° clockwise from vertical, and the other half were 24° < Δ ≤ 36° apart from 45° (Δ). The number of 45° words was counterbalanced across the four subsets of 10 stimuli differing in their syllable counts and semantic category. Thus, in each subset, five words were at 45° and the others were deviant from 45°. To prevent participants from estimating the number of syllables in a word from its visual length, each word was padded with the hashtag symbol(s) and displayed in the monospaced font Courier New to give all stimuli an equal length. Each word's location varied randomly across trials in the range of −9.4 cm and 9.4 cm horizontally from the centre of the monitor. Like Experiment 1, this measure was taken to prevent participants from using a tool to help judge the orientation of a word. The visual words were generated using a customised MATLAB script.

#### Procedure

**Figure 6.**
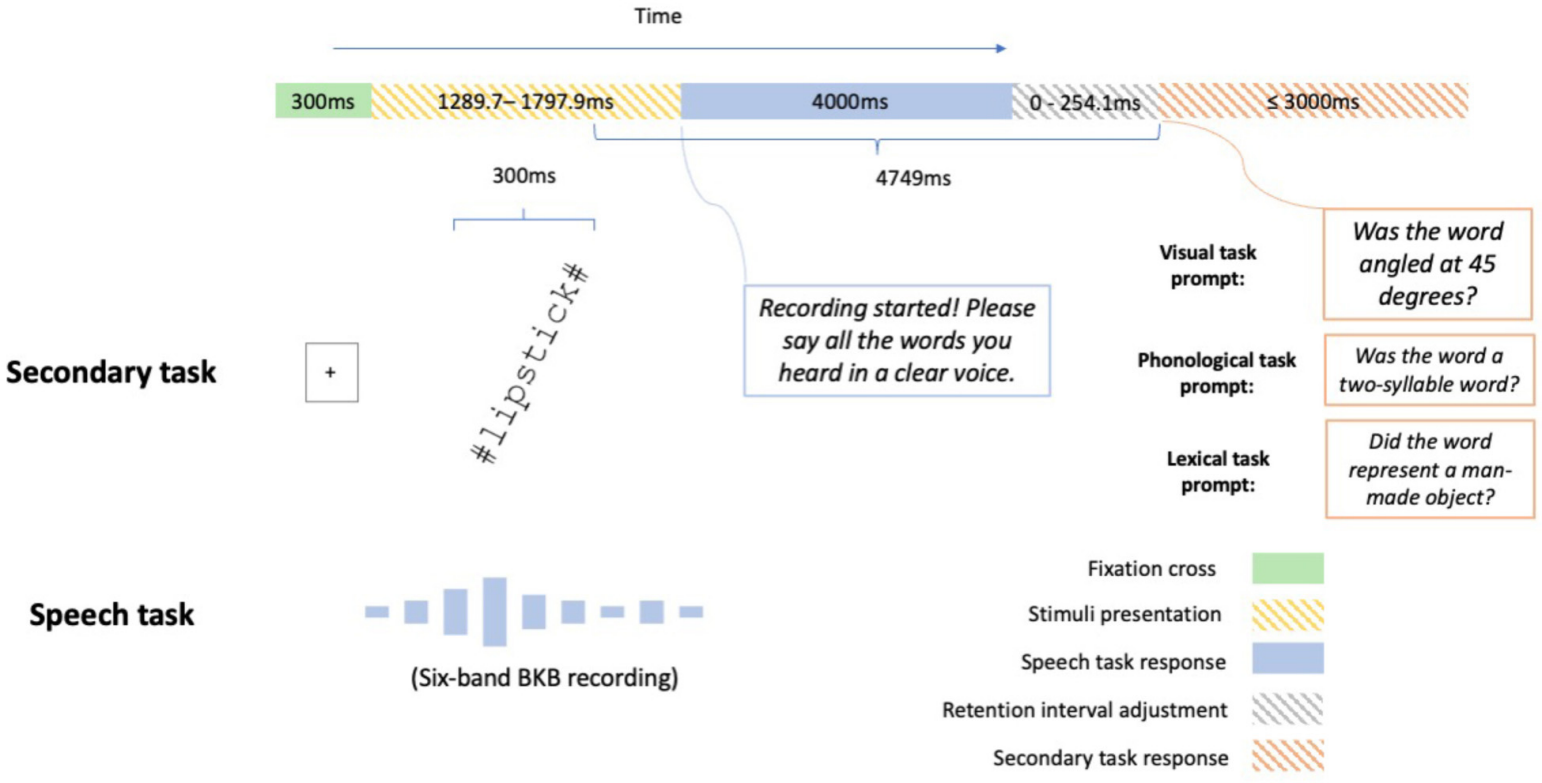
In Experiment 2, participants recognised a six-band Bamford Kowal-Bench (BKB) sentence while they saw a visual word flashing briefly. All secondary task conditions used the same set of stimuli. Participants in the visual task condition decided whether the word was oriented at 45° clockwise from vertical. Participants who did the phonological task judged whether the word (e.g., camera) was a two-syllable word. Those who performed the lexical task decided whether the word (e.g., kangaroo) was a man-made object. Like in Experiment 1, a retention interval adjustment was added between the responses to the speech task and the secondary task. The plots of the fixation cross and the visual word are for illustration only and not scaled to the actual size.

The procedure of Experiment 2 was similar to Experiment 1, with the following differences. First, participants in Experiment 2 were not allowed to continue the experiment if their monitor size was smaller than 12.1 inches or display resolution was less than 1440*900. This stipulation was included because the horizontal dimension of a monitor smaller than 12.1 inches (with a typical 16:10 ratio) was shorter than that of the stimulus image (22.6 cm), hence the visual stimuli would have been scaled to smaller than their desired size. Second, the Gabor stimuli for the main dual-task were replaced by visual words. In Experiment 2, participants in a between-group design recognised a noise-vocoded sentence while performing either a visual, phonological or a lexical secondary task (Figure 6). Participants responded by pressing the left (‘Yes’) or right (‘No’) arrow keys. Half of the trials had a correct answer of ‘Yes’. The experiment took 24 min [*SD* = 13.68 min] on average.

#### Dependent Measures

In Experiment 1, we measured % of correctly recognised key words in the speech task. We also measured the response correctness and RTs for the secondary task. Ratings on effort and attention invested in the dual task were collected after the main task.

#### Analysis

A set of three GLMMs was fit for Experiment 2 on the observed proportion of correct key words in the speech task, as well as response correctness and RTs in the secondary tasks to assess whether trial and task conditions modulated these performance measures. All models contained Task, Trial, and their interaction as predictors and included a logarithmic transformation on Trial to account for the non-linear trend of perceptual learning (see Supplemental Table B1 for a comparison between the model fit for transformed and non-transformed predictors). The Task predictor included levels of single, visual, phonological and lexical for speech % correct, and visual, phonological and lexical for the secondary-task correctness and RTs. The GLMMs were first fitted to the maximal random-effect structures that were identical to those in Experiment 1 and underwent the same model selection procedure. The final models on speech % correct and secondary task correctness included random intercepts for the Participant and random slopes for Trial by Participant. The model on secondary task RT included random intercepts for Prompt and random slopes for Trial by Prompt.

### Experiment 2: Results

#### Speech Task

**Figure 7.**
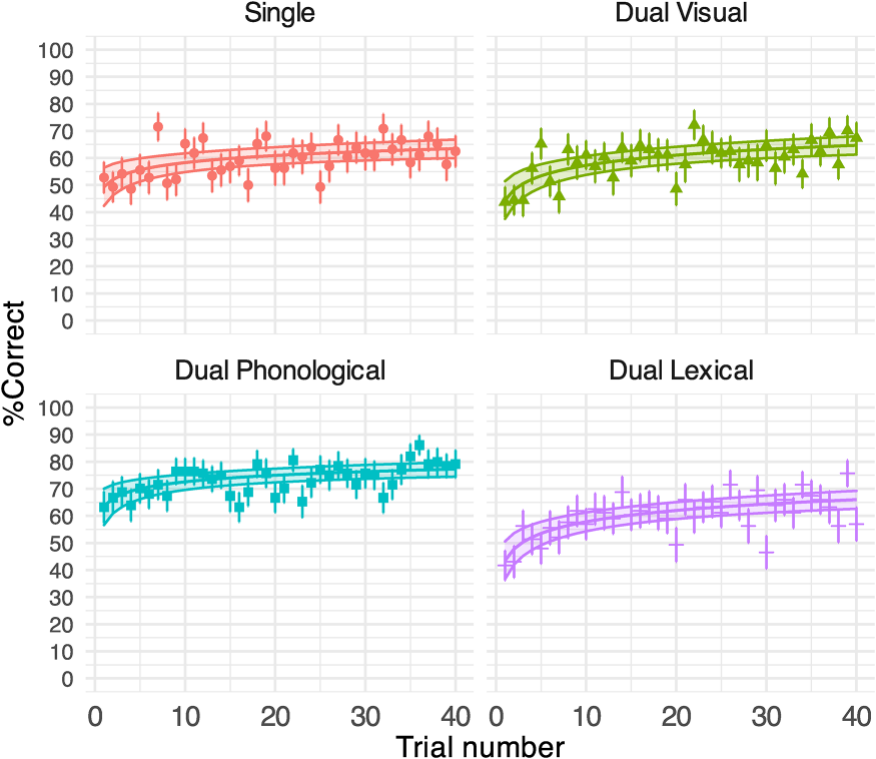
Generalised linear mixed-effect model (GLMM)-estimated percent of correctly reported key words in Experiment 2 displayed as a function of the trial (middle solid lines in the coloured areas). Each panel illustrates the results under each task condition. Filled areas represent 95% confidence intervals. Points denote the raw mean % correct obtained on each trial. Error bars indicate the standard error of the mean. Raw means and error bars for the single speech task were re-plotted from Experiment 1.

Table 2 shows the GLMM outputs. Figure 7 shows the % correct of sentence recognition per Trial per Task and the GLMM predictions. The overall speech % correct were comparable under the lexical and visual secondary tasks (60.03% [*SD* = 12.07] vs. 59.44% [*SD* = 9.34]), as well as the single speech task (59.57% [*SD* = 11.71]). However, the speech performance was significantly higher for the phonological task than all other conditions (73.26% [*SD* = 8.58], Table 2). Trial significantly affected sentence recognition comparably in all conditions (Table 2; all *p*'s < .001 for log(trial) terms, non-significant interaction terms between Trial and Task conditions). Figure 7 shows that these effects came from a significant improvement in speech % correct over 40 trials in all groups: single (14.08%), dual visual (20.31%), dual phonological (13.66%) and dual lexical (22.53%).

**Table 2.** Model Outputs for the GLMM Assessing the Fixed Effects of Task and Trial on the Speech Task Accuracy in Experiment 2.

Fixed effects				
	*β*	*SE*	*z*	*p*
(Intercept)	0.56	0.15	3.75	**<**.**001**
log(trial)	0.18	0.05	3.95	**<**.**001**
dual_lexical [dual_phonological]	−0.82	0.21	−3.95	**<**.**001**
speech_single [dual_phonological]	−0.58	0.21	−2.81	.**005**
dual_visual [dual_phonological]	−0.78	0.21	−3.78	**<**.**001**
log(trial):dual_lexical [dual_phonological]	0.07	0.06	1.12	.264
log(trial):speech_single [dual_phonological]	−0.02	0.06	−0.38	.706
log(trial):dual_visual [dual_phonological]	0.05	0.06	0.71	.476

Abbreviations: GLMM = generalised linear mixed-effect model; *SE* = standard error.

The reference level is shown in a bracket.

#### Secondary Task

**Figure 8.**
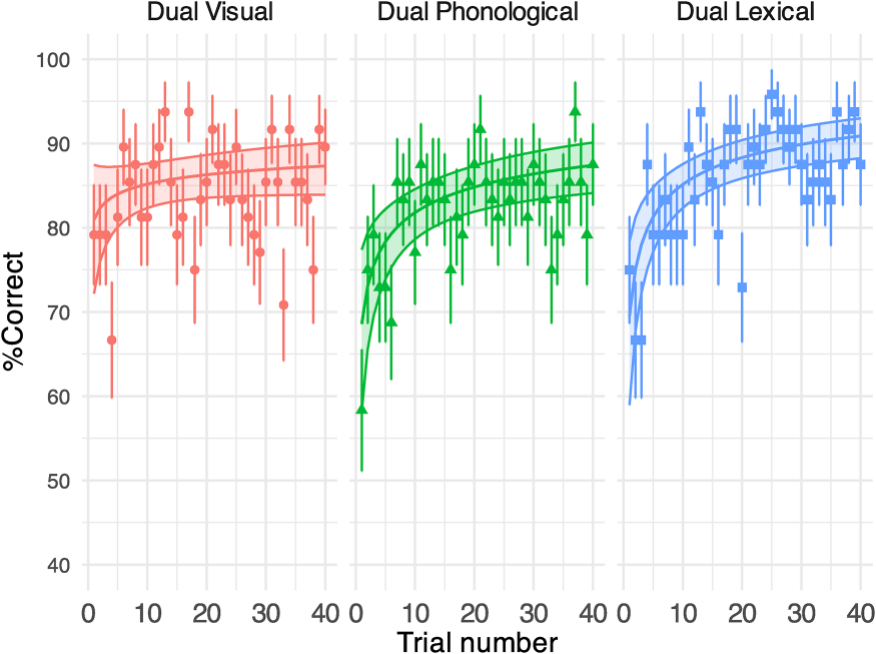
Generalised linear mixed-effect model (GLMM)-estimated percent of correct responses for the secondary task in Experiment 2, displayed as a function of the trial (middle solid lines in the coloured areas). Each panel illustrates the results under each task condition. Filled areas represent 95% confidence intervals. Points denote the raw % correct of response (i.e., number of correctly responded participants/total number of participants * 100) on each trial. Error bars indicate standard error of the accuracy.

**Figure 9.**
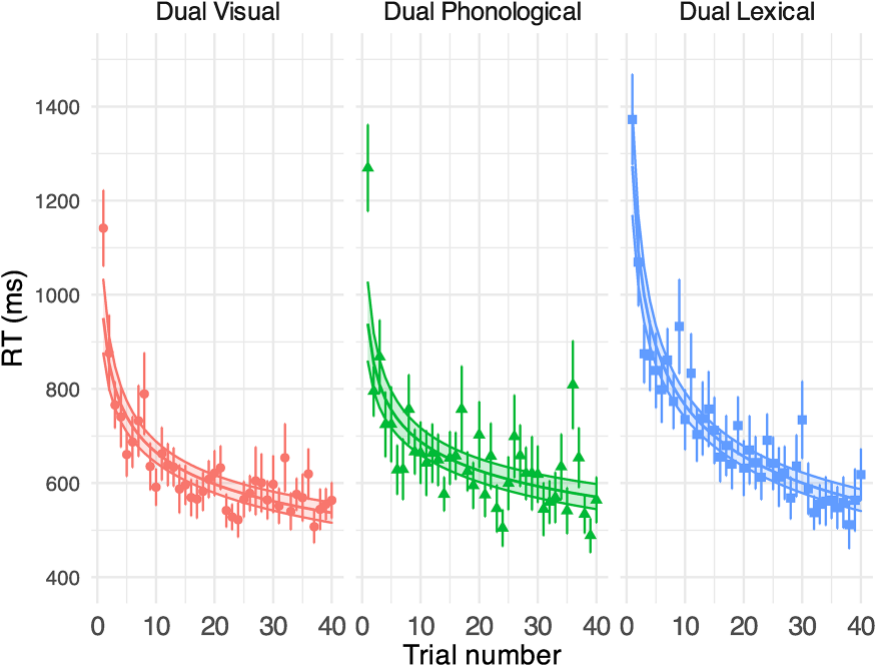
GLMM-estimated visual task RTs in milliseconds in different secondary tasks in Experiment 2, displayed as a function of the trial (middle solid lines in the coloured areas). Each panel illustrates the results under each task condition. Filled areas represent 95% confidence intervals. Points denote the raw mean RTs for correct secondary task responses obtained on each trial. Error bars indicate the standard error of the mean. See Supplemental Table B6 for model output.

Abbreviations: GLMM = generalised linear mixed-effect model; RTs = response times.

Figure 8 shows the response correctness (displayed in % correct) in performing the secondary tasks (see Supplemental Table B5 for model outputs). The task performance was above chance level (0.5) in all conditions – visual (84% [*SD* = 11]), phonological (82% [*SD* = 10]) and lexical (86% [*SD* = 7]). The accuracy was similar across tasks. Percent correct did not improve through the session of the visual task, but significantly increased over the course of the phonological and the lexical task at a similar rate. See Figure 9 for the RT results.

### Experiment 2: Discussion

In Experiment 2, we observed speech perceptual learning under all three dual-task conditions comparable to the baseline single speech condition (Figure 7), suggesting that adapting to noise-vocoded speech might not rely on the type of domain-general (visual) and domain-specific (phonological or lexical) processes required by the secondary task.

Therefore, our results supported Hypothesis 1 – perceptual learning of noise-vocoded speech does not strictly require domain-general or domain-specific resources. The inclusion of a secondary task did not modulate speech perceptual learning compared with the baseline task. Hypothesis 2 – perceptual learning of speech depends on domain-general, rather than domain-specific processes, was rejected, as learning under the visual task was similar to that in the single task, and learning under the language tasks was no worse than that observed in the visual task. Hypothesis 3 – perceptual learning of speech relies critically on language processes in general, was not supported, as overall improvement in the phonological or lexical condition did not differ from the visual condition. Hypothesis 4 – lexical processing is more important than phonological processing for perceptual learning – was not supported either. Thus, our results showed that distraction due to divided attention in visual, phonological or lexical processes does not affect the course of speech-perceptual learning.

## General Discussion

### Speech Perceptual Learning Under Divided Attention

In two experiments, we explored whether speech perceptual learning is a function of the availability of attentional resources (Experiment 1) and if and how learning is affected by distraction in different aspects of mental processes (Experiment 2). Despite divided attention and more effortful speech processing (Supplemental Figures C4 and C5), perceptual learning was intact compared to the single speech task under an easy and intermediate visual task (Experiments 1 and 2), as well as a phonological and a lexical task. (Experiment 2), where the amount and trend of speech perceptual learning were not modulated by performing the secondary task. Although speech performance was suppressed in early trials under the most difficult visual condition in Experiment 1, listeners achieved quicker learning – thus an ending performance comparable to the single speech condition. Our results illustrated that perceptual learning remains robust under different magnitudes (Experiment 1) and types (Experiment 2) of divided attention. These findings contributed to the current theories by showing that undivided attention is not necessary for speech-perceptual learning and that learning is resilient to distraction that is either domain-general or domain-specific to speech processing.

For the easy and hard visual tasks in Experiment 1 and the phonological and lexical tasks in Experiment 2, the % correct improved dramatically in early trials (i.e., Figures 4 and 8). As such, the resources devoted to the secondary tasks may not have been consistent across trials (e.g., less resources on secondary tasks in later trials), which could have contributed to the observed learning of speech. It is noteworthy though, that speech task performance improved dramatically under all dual-task conditions even during the phase when the secondary task was likely to exert a significant processing load (i.e., early trials 1–15), which signalled that perceptual learning of speech was resilient to divided attention. In both experiments, the inconsistent secondary task load over trials did not seem to affect the overall course of speech perceptual learning at a behavioural level. Learning in the speech task under all dual-task conditions showed a similar trend compared to the single speech condition except for under the hard visual condition. Importantly, the individual slope of speech perceptual learning (i.e., the individual beta estimate for the log(trial) term) did not predict improvements in the secondary task (see Supplemental Figure C6 for additional details, and Supplemental Appendix E for a link to the analysis script). Therefore, the patterns of learning observed for the speech task were not likely a by-product of secondary task learning. However, a future variation to the current setup may be to first train people on the secondary tasks, to avoid task learning on the secondary task during the dual task, before using those tasks to provide a consistently difficult secondary task.

Because perceptual learning remained unaffected until the secondary task exerted a heavy load on speech processing, it is possible that the effect of inattention on the perceptual learning of sentences is related to whether speech stimuli were actively attended to. A study by Mukai et al. (2011) observed perceptual learning of low-level visual orientation under divided attention and focused attention but not when participants did not attend to the target stimuli and performed a task that only engaged the processing of the distractors. Mukai et al.'s findings suggest that exhausting attention by performing a background task eliminates perceptual learning. Perceptual learning may not occur when participants passively listen to speech while their attention is exhausted by performing an external task. In contrast, perceptual learning can take place when participants’ attention is divided by concurrent tasks. This distinction between attending to one task with other stimuli presented in the background and dividing attention between two tasks might explain why Huyck and Johnsrude (2012) did not observe learning for unattended noise-vocoded speech, and why we found perceptual learning under divided attention.

Speech perceptual learning during the early trials of the most difficult visual task had a larger gradient (i.e., a steeper slope). The challenging visual task impeded speech recognition only when listeners were first exposed to the dual task setup but did not constrain the amount of learning. This pattern of results is corroborated by Banks et al. (2015a), where listeners with lower starting accuracy adapted more to the accented speech. It seems that perceptual learning in the current experiments served as a mechanism that reduces the impact of distraction on speech processing (Shiffrin & Schneider, 1977). Fast learning in the first few trials in a challenging dual-task brought the speech perception performance to the single-task level. Therefore, the overall speech performance was comparable to the single task even under challenging conditions.

### Roles of Attention in Rapid and Long-Term Perceptual Learning of Speech

The current experiments addressed the role of attention in rapid perceptual learning of noise-vocoded speech during a short exposure (40 sentences). For all task conditions, speech recognition continued to improve throughout the session, leaving open the possibility that performance would improve further if a longer task or a multi-session study were administered. On the other hand, in Rosen et al. (1999), a study exploring the perceptual learning of six-band BKB sentences in a longer training paradigm, the authors found a 15% improvement in word report over 10 sessions (a total of 384 sentences), which is comparable to the magnitude of learning in the current study (12.61%). Therefore, learning in the current studies seems to be fast during a short exposure and might have the potential to complete shortly thereafter.

However, it is possible that divided attention has a different impact on long-term perceptual learning than the rapid adaptation we explored. A recent study in the visual domain found that directing attention to the task-relevant spatial cue during a one-week training for a visual orientation discrimination task significantly facilitated the transfer learning of the same task in an untrained location in the visual field (Hung & Carrasco, 2021). This benefit lasted for around one year after the training period, showing a long-term effect of attention on visual plasticity. As such, it remains an open question whether attention can affect the training outcome over a long period and provide long-lasting benefits to perceptual learning of degraded speech.

### Enhanced Speech Processing Under a Phonological Task

We also found greater sentence recognition performance in the phonological condition than in all other conditions in Experiment 2. One possible explanation is that we happened to select a group of low-performing listeners for our single speech task. However, there was no detectable difference in % correct between the current baseline condition and our pilot testing (*n* = 30, 60 trials) in a binomial random-intercept model (59.57% [*SD* = 11.71] vs. 58.61% [*SD* = 10.62], see Supplemental Figure C3), so this possibility does not seem likely. Moreover, we also replicated the phonological dual task with 48 new participants (Supplemental Appendix C). The speech performance was almost equivalent under the replicated and original tasks (72.81% [*SD *= 9.49] vs. 73.26% [*SD* = 8.58]) whose difference was not detectable by GLMM (*p* = .956; Supplemental Figure C2). This replication showed a robust effect of Trial on speech perception [*β* (*SE*)* =* 0.173 (0.048), *p* < .001] and produced a 13.13% improvement which was comparable to the original task (13.84%). Moreover, the phonological task in the replication achieved an accuracy closely resembling that of the original task (82% [*SD* = 10] vs. 82% [*SD* = 9]; Supplemental Figure C3). As such, this replication closely matched the results of the original phonological condition in Experiment 2. It, therefore, seems plausible that speech processing under the phonological condition was enhanced by performing the concurrent task.

Another explanation considers the interaction of the processes required by each task in a dual-task context. Kim et al. (2005) illustrated in a dual task that Stroop interference (Stroop, 1935), the delay in RT between congruent and incongruent stimuli, increased when the secondary task (i.e., character detection) overlapped with the target processing (i.e., verbal processing) in the main task – comparing literal meanings of coloured words. In contrast, the same effect decreased when the secondary task required a process (i.e., verbal process) that distracted the target processing (i.e., visuospatial processing) in the main task – comparing ink colours of words of colour. These findings suggest that interference of the secondary task with a process unnecessarily or even hindering the main task can boost task performance for the main task. Thus, the phonological secondary task in our Experiment 2 might have affected the balance between the top-down and bottom-up processes in tuning perception. In other words, occupying the phonological speech process with the secondary task might have facilitated degraded speech processing, by directing attention to the task-relevant lexical information.

While the behavioural results for the visual and lexical tasks were both similar to the single speech condition, we cannot exclude the possibility that distinct cognitive and/or neural mechanisms support speech processing for visual or lexical tasks. For example, a lexical task might facilitate brain activity in areas related to lexical-level semantic processing (e.g., inferior frontal gyrus; Sohoglu & Davis, 2016; Zekveld et al., 2012). In contrast, a visual task might result in elevated activities in brain regions associated with visual processing (e.g., lateral occipital cortex; Gennari et al., 2018) and attentional control (e.g., anterior cingulate cortex, primary angle-closure glaucoma; Gennari et al., 2018), which will reflect general task load for processing the visual word inputs.

### Limitations

We only included 40 trials in both experiments to prevent fatigue in online participants. However, speech perceptual learning seemed to be incomplete in several task conditions (e.g., single-task condition and the easier Gabor conditions), because there was a linear trend of improving performance towards the end of the task. Therefore, perhaps longer exposure with more trials (e.g., Mukai et al., 2011; Trotter et al., 2021) would have been useful. Exposure to more sentences might have revealed later-stage learning differences between the single and dual-task conditions. It should be stressed, however, studies tracking the time course of the perceptual learning of moderately degraded noise-vocoded speech (e.g., four or six bands; Cooke et al., 2022; Erb et al., 2013) illustrated that the largest amount of learning happens in the first 10 trials, which is covered in the current study.

Moreover, despite the results of Experiment 2 showing boosted speech processing under a concurrent phonological task, this effect is subject to further investigation. Future studies could consider adding time-compressed speech to the design (i.e., a faster presentation of speech than the normal rate). The successful recognition of this type of speech is thought to rely on pre-lexical, phonological processing (Pallier et al., 1998; Sebastián-Gallés et al., 2000). Therefore, if the secondary task can indeed facilitate speech processing by occupying a less relevant mental process, the lexical and the phonological task should have a different impact on speech processing depending on the type of degradation. Per predictive coding, a phonological task should facilitate the processing of noise-vocoded speech but hinder the perception of time-compressed speech, whereas the lexical task is predicted to enhance the performance for time-compressed speech but hamper the processing of noise-vocoded speech. This is because the lexical process outweighs the phonological process in perceiving noise-vocoded speech, and vice versa for the time-compressed speech.

Finally, age and hearing loss can also affect the impact of divided attention on speech perceptual learning. The current study only recruited young listeners with a normal hearing capacity. However, past evidence has shown that listeners with hearing loss have reduced spectrotemporal and spatial acuity (e.g., Bernstein & Oxenham, 2006; Cusack et al., 2004; Drennan et al., 2003) and show lower ability to use these cues for selectively attending to a target signal (e.g., a target talker among competing talkers; e.g., Best et al., 2009; Dai et al., 2018; Roverud et al., 2020). Moreover, perceptual learning seems to diverge in young and old listeners. For example, during rapid perceptual learning of time-compressed speech, both groups showed comparable magnitude of learning, but old listeners failed to show additional benefit with a longer training phase and did not show transfer of the learning to a different rate of speech (Peelle & Wingfield, 2005). Together, these studies insinuate that speech perceptual learning and its relationship with attention might differ with age and hearing loss. It remains to be seen if and how divided attention impact on the perceptual learning of noise-vocoded speech in older and/or hearing-impaired adults.

## Conclusion

We examined the role of divided attention in the perceptual learning of noise-vocoded speech. Speech perceptual learning with a concurrent visual task remained comparable to the single speech task up to the point the secondary task exerted a heavy load on speech processing, where listeners demonstrated faster learning. We also showed that speech perceptual learning does not strictly require domain-general or domain-specific resources: perceptual learning under a visual, phonological, and lexical secondary task was as robust as under a single speech task. Overall, our results show that the effect of divided attention on rapid perceptual learning of a short exposure to speech is not dependent on the domain-specificity of the secondary task. Our results clarify current theoretical accounts (Amitay, 2009; Feldman & Friston, 2010; Goldstone, 1998) by demonstrating that undivided attention is not required for rapid perceptual learning of speech.

## Supplemental Material

sj-pdf-1-tia-10.1177_23312165231192297 - Supplemental material for Perceptual Learning of Noise-Vocoded Speech Under Divided Attention Click here for additional data file. Supplemental material, sj-pdf-1-tia-10.1177_23312165231192297 for Perceptual Learning of Noise-Vocoded Speech Under Divided Attention by Han Wang, Rongru Chen, Yu Yan, Carolyn McGettigan, Stuart Rosen and Patti Adank in Trends in Hearing
