# Supplementary material for: Perceptual Learning of Noise-Vocoded Speech Under Divided Attention
Source: Trends Hear. 2023 Aug 7;27:23312165231192297. doi: 10.1177/23312165231192297 (PMC10408355; doi:10.1177/23312165231192297)
Supplement: sj-pdf-1-tia-10.1177_23312165231192297 - Supplemental material for Perceptual Learning of Noise-Vocoded Speech Under Divided Attention [file sj-pdf-1-tia-10.1177_23312165231192297.pdf]

Appendix A

Sentence and word stimuli used in the experiments

Table A1

The sentence stimuli of the speech task (Experiments 1 and 2) and the word stimuli of the secondary task (Experiment 2)

Sentence (speech task)	Keyword 1	Keyword 2	Keyword 3	Word (secondary task)
The postman shut the gate.	postman	shut	gate	guitar
The ladder's near the door.	ladder’s	near	door	cupboard
The old gloves are dirty.	old	gloves	dirty	scissors
The thin dog was hungry.	thin	dog	hungry	statue
The police chased the car.	police	chased	car	boiler
Some sticks were under the tree.	sticks	under	tree	lipstick
The school finished early.	school	finished	early	headphones
The man cleaned his shoes.	man	cleaned	shoes	lighthouse
The jam jar was full.	jam	jar	full	slipper
The cook’s making a cake.	cook’s	making	cake	turbine
The bath towel was wet.	bath	towel	wet	container
The matches lie on the shelf.	matches	lie	shelf	motorbike
Somebody took the money.	somebody	took	money	pyjamas
The mother stirs the tea.	mother	stirs	tea	sunglasses
The broom stood in the corner.	broom	stood	corner	trampoline
The orange was quite sweet.	orange	quite	sweet	microphone
The match boxes are empty.	match	boxes	empty	computer
The bus stopped suddenly.	bus	stopped	suddenly	detector
A letter fell on the mat.	letter	fell	mat	camera
The lady packed her bag.	lady	packed	bag	basketball
The dinner plate's hot.	dinner	plate’s	hot	dolphin
Someone’s crossing the road.	someone’s	crossing	road	penguin
The front garden was pretty.	front	garden	pretty	giraffe
The taps are above the sink.	taps	above	sink	lizard
The bread van's coming.	bread	van’s	coming	hamster
The children help the milkman.	children	help	milkman	reindeer
The boy had a toy dragon.	boy	toy	dragon	tortoise
The fruit came in a box.	fruit	came	box	leopard
The kitchen window was clean.	kitchen	window	clean	hedgehog
The grass is getting long.	grass	getting	long	rhino
The puppy plays with a ball.	puppy	plays	ball	octopus
Some men shave in the morning.	men	shave	morning	kangaroo
The painter used a brush.	painter	used	brush	jellyfish

The apple pie's cooking.	apple	pie’s	cooking	woodpecker
The cleaner swept the floor.	cleaner	swept	floor	antelope
A grocer sells butter.	grocer	sells	butter	ladybird
The raincoat's hanging up.	raincoat’s	hanging	up	chimpanzee
The flower stands in a pot.	flower	stands	pot	mosquito
The egg cups are on the table.	egg	cups	table	dragonfly
The cricket team's playing.	cricket	team’s	playing	cockerel

Appendix B

Supplementary model outputs

Table B1

Model comparisons for the goodness-of-fit (AIC) of the fixed-effect generalised linear models with or without a transformation to the Trial predictor. Each model had Trial, Task, and their Interaction as predictors. P value was obtained using $p = \exp\left(\frac{-|AIC_{model1} - AIC_{model2}|}{2}\right)$ *from Burnham & Anderson (2004).*

Comparison	Model	AIC	*p*
1	m_exp1_speech_glm	24600	
	m_exp1_speech_glm_log	24570	**< 0.001**
2	m_exp1_visual_accuracy_glm	6390	
	m_exp1_visual_accuracy_poly	6379	**< 0.001**
3	m_exp1_visual_rt_glm	59150	
	m_exp1_visual_rt_glm_log	59070	**< 0.001**
4	m_exp2_speech_glm	23940	
	m_exp2_speech_glm_log	23910	**< 0.001**
5	m_exp2_secondary_accuracy_glm	5057	
	m_exp2_secondary_accuracy_glm_log	5041	**< 0.001**
6	m_exp2_secondary_rt_glm	68680	
	m_exp2_secondary_rt_glm_log	68570	**< 0.001**

Table B2

Model outputs for the GLMM assessing the fixed effects of Task and Trial on the visual task accuracy in Experiment 1. The reference level is shown a bracket.

**Fixed effects:**				
	β	Std. Error	*z*	*p*
(Intercept)	1.24	0.07	16.56	**< 0.001**
poly(trial, 2)1	-0.74	4.04	-0.18	0.854
poly(trial, 2)2	-9.28	4.58	-2.03	**0.043**
dual_hard [dual_intermediate]	-0.79	0.10	-7.79	**< 0.001**
dual_easy [dual_intermediate]	0.44	0.11	4.04	**< 0.001**
poly(trial, 2)1:dual_hard [dual_intermediate]	1.41	5.42	0.26	0.794
poly(trial, 2)2:dual_hard [dual_intermediate]	5.44	6.22	0.87	0.382
poly(trial, 2)1:dual_easy [dual_intermediate]	7.52	6.04	1.25	0.213
poly(trial, 2)2:dual_easy [dual_intermediate]	-5.09	6.83	-0.75	0.456

Table B3

Model outputs for the GLMM assessing the fixed effects of Task and Trial on the visual task RT in Experiment 1. The reference level is shown a bracket.

**Fixed effects:**				
	β	Std. Error	*z*	*p*
(Intercept)	6.90	0.06	110.27	**< 0.001**
log(trial)	-0.16	0.01	-11.43	**< 0.001**
dual_intermediate [dual_hard]	-0.34	0.09	-3.88	**< 0.001**
dual_easy [dual_hard]	-0.26	0.09	-2.97	**0.003**
log(trial):dual_intermediate [dual_hard]	0.05	0.02	2.90	**0.004**
log(trial):dual_easy [dual_hard]	0.04	0.02	2.25	**0.024**

Table B4

Model outputs for the simple linear regression (LM) assessing effects of Task condition on participants' estimated effort and attention in Experiment 1. The reference level for the predictor is shown in brackets. The models included Task as a predictor and Participant Rating as response. Four separate models were fitted to the effort and attention ratings in the speech and visual tasks. These models did not include random effect as there was only one observation per participant per question per Task.

model	coefficients	*β*	Std. Error	*t*	*p*
effort_speech	(Intercept)	66.15	2.69	24.57	**< 0.001**
	dual hard [single]	12.85	3.83	3.36	**0.001**
	dual intermediate [single]	12.04	3.81	3.16	**0.002**
	dual easy [single]	12.49	3.83	3.26	**0.001**
attention_speech	(Intercept)	96.31	1.47	65.43	**< 0.001**
	dual hard [single]	-3.21	2.06	-1.56	0.121
	dual intermediate [single]	-3.90	2.07	-1.88	0.061
	dual easy [single]	-2.44	2.07	-1.18	0.240
effort_visual	(Intercept)	65.02	3.87	16.81	**< 0.001**
	dual hard [easy]	13.48	5.47	2.46	**0.015**
	dual intermediate [easy]	8.26	5.50	1.50	0.136
attention_visual	(Intercept)	76.19	3.41	22.38	**< 0.001**
	dual hard [easy]	-2.25	4.82	-0.47	0.641
	dual intermediate [easy]	3.54	4.82	0.74	0.463

Table B5

Model outputs for the GLMM assessing the fixed effects of Task and Trial on the secondary task accuracy in Experiment 2. The reference level is shown a bracket.

**Fixed effects:**				
	β	Std. Error	*z*	*p*
(Intercept)	0.82	0.24	3.49	**< 0.001**
log(trial)	0.40	0.09	4.70	**< 0.001**
dual_phonological [dual_lexical]	-0.04	0.32	-0.14	0.892
dual_visual [dual_lexical]	0.62	0.34	1.85	0.065
log(trial):dual_phon [dual_lexical]	-0.09	0.12	-0.74	0.459
log(trial):dual_visual [dual_lexical]	-0.27	0.12	-2.24	**0.025**

Table B6

Model outputs for the GLMM assessing the fixed effects of Task and Trial on the secondary task RT in Experiment 2. The reference level is shown a bracket.

**Fixed effects:**				
	β	Std. Error	*z*	*P*
(Intercept)	7.15	0.04	162.17	**< 0.001**
log(trial)	-0.22	0.01	-15.15	**< 0.001**
dual_phonological [dual_lexical]	-0.30	0.06	-4.86	**< 0.001**
dual_visual [dual_lexical]	-0.29	0.06	-4.86	**< 0.001**
log(trial):dual_phon [dual_lexical]	0.09	0.02	4.17	**< 0.001**
log(trial):dual_visual [dual_lexical]	0.07	0.02	3.27	**0.001**

Table B7

Model outputs for the LM assessing effects of Task condition on participants' estimated effort and attention in Experiment 2. The reference level for the predictor is shown in brackets.

model	coefficients	*β*	Std. Error	*t*	*p*
effort_speech	(Intercept)	66.15	2.53	26.13	**< 0.001**
	dual lexical [single]	14.25	3.62	3.94	**< 0.001**
	dual phonological [single]	3.59	3.62	0.99	0.322
	dual visual [single]	17.45	3.60	4.85	**< 0.001**
attention_speech	(Intercept)	96.31	1.23	78.13	**< 0.001**
	dual lexical [single]	-3.46	1.73	-2.01	**0.046**
	dual phonological [single]	-3.89	1.73	-2.25	**0.026**
	dual visual [single]	-1.03	1.73	-0.59	0.554
effort_secondary	(Intercept)	71.90	4.32	16.66	**< 0.001**
	dual lexical [visual]	-14.58	6.10	-2.39	**0.018**
	dual phonological [visual]	-7.25	6.10	-1.19	0.237
attention_secondary	(Intercept)	67.21	3.35	20.05	**< 0.001**
	dual lexical [visual]	10.19	4.74	2.15	**0.033**
	dual phonological [visual]	13.92	4.74	2.94	**0.004**

Appendix C

Supplementary figures

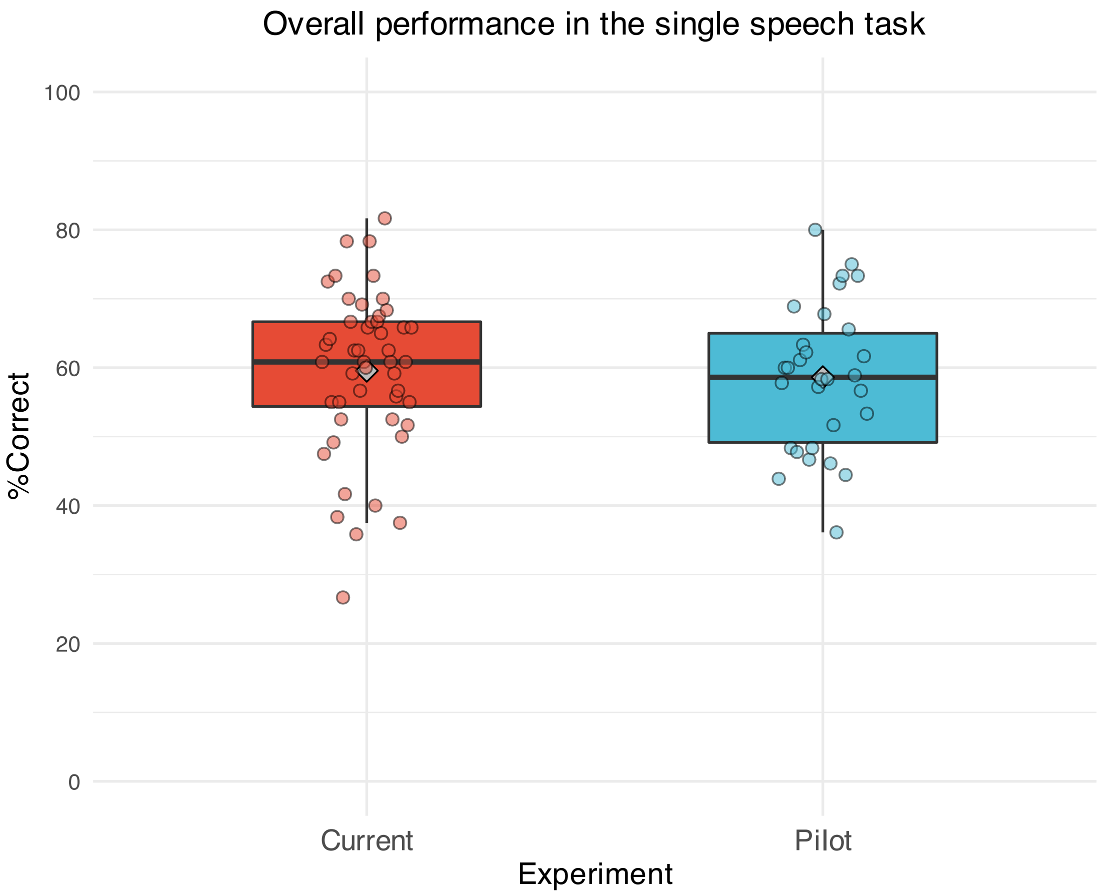

Figure C1. Percent of correctly reported key words in the single speech task of the current (red box) and the pilot (blue box) experiments. Points display the raw mean % correct of speech responses per participant. Grey diamonds denote the group mean of each condition.

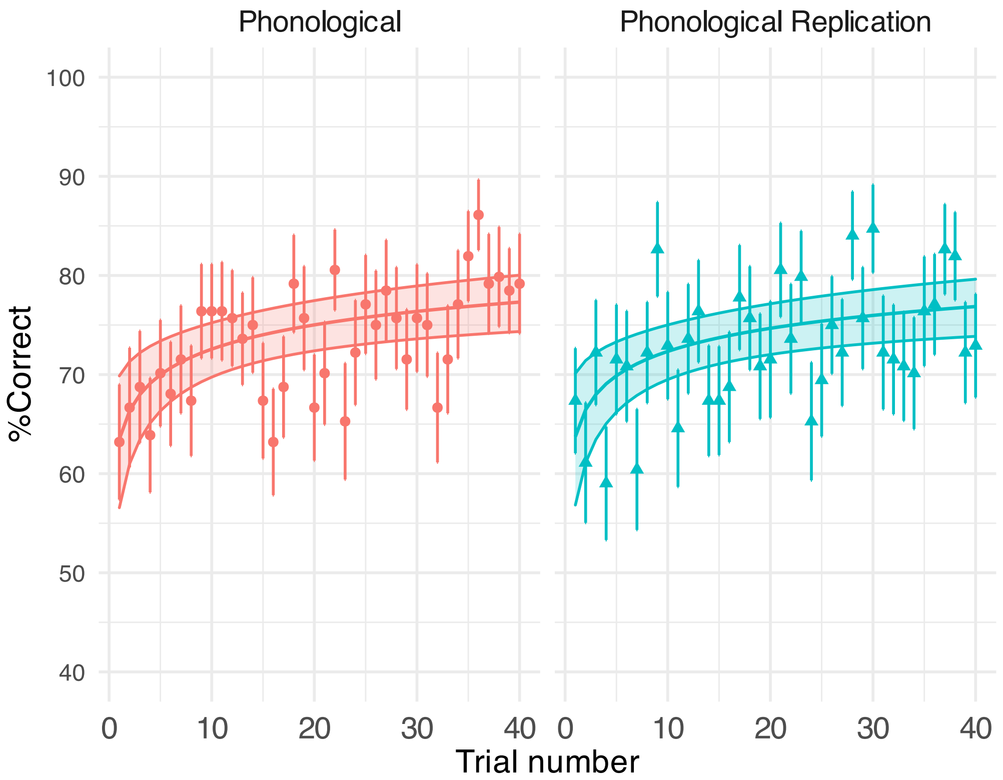

Figure C2. GLMM-estimated percent of correctly reported key words in the original and replaced phonological conditions displayed as a function of trial (middle solid lines in the coloured areas). Each panel illustrates the results under each task condition. Filled areas represent 95% confidence intervals. Points denote the raw mean % correct obtained on each trial. Error bars indicate standard error of the mean.

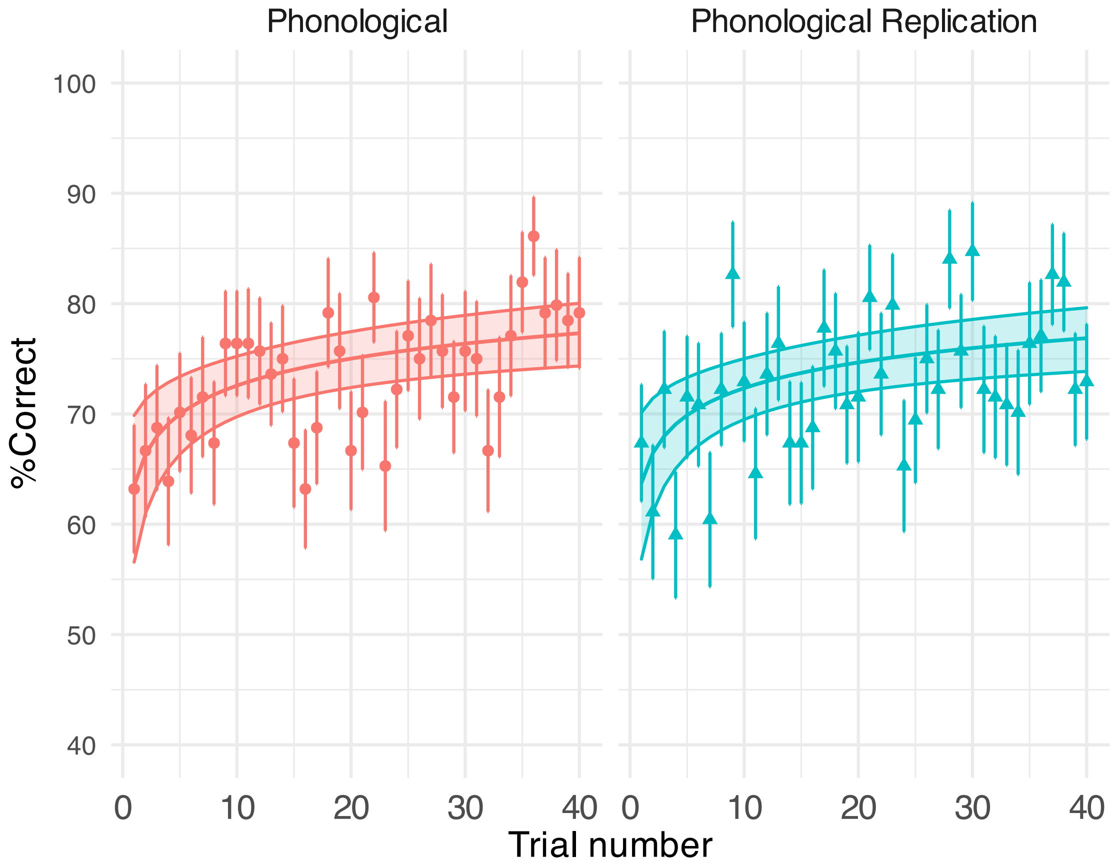

Figure C3. GLMM-estimated secondary task accuracy in the original and replaced phonological conditions, displayed as a function of trial (middle solid lines in the coloured areas). Each panel illustrates the results under each task condition. Filled areas represent 95% confidence intervals. Points denote the raw accuracy of response (i.e., number of correctly responded participants / total number of participants) on each trial. Error bars indicate standard error of the mean accuracy. In both tasks, Trial significantly modulated performance (phonological [β *(SE)* = 0.316 (0.078), $p < .001$], phonological replication [β *(SE)* = 0.199 (0.081), $p = .013$]), where the accuracy of response improved over time. However, the overall accuracy was not different between the two tasks [β *(SE)* = 0.320 (0.311), $p = .303$].

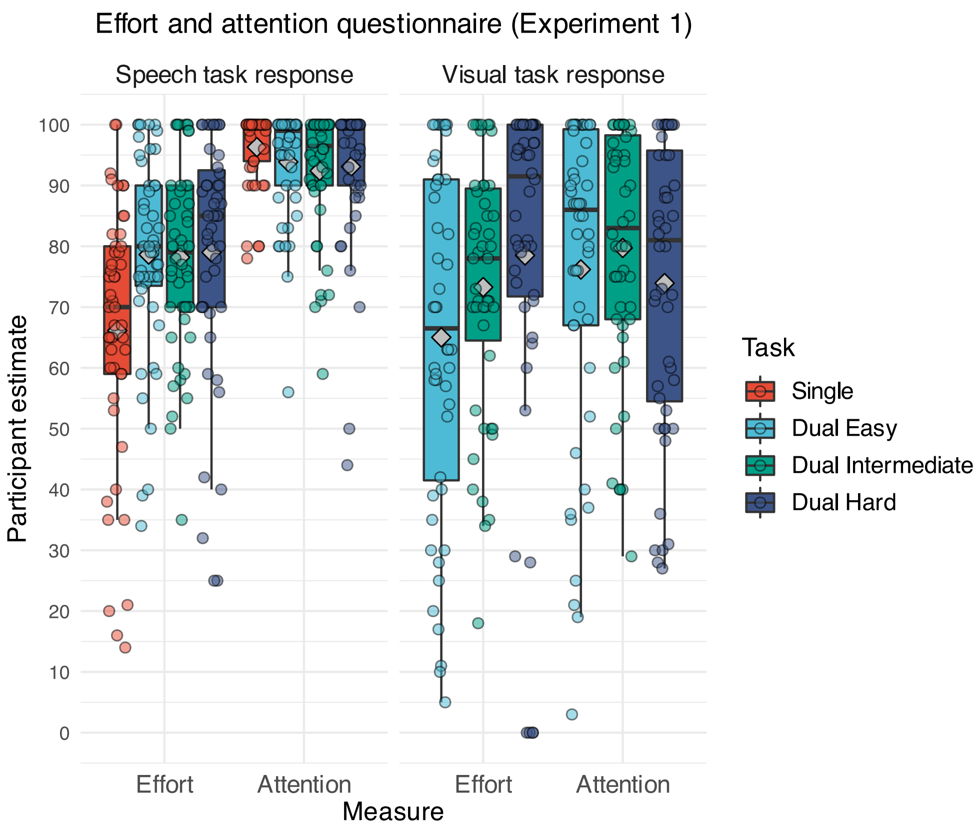

Figure C4. Participants' estimate on their effort and attention invested in the speech and visual task under different task conditions in Experiment 1. The fill of the boxes represents task condition. Each panel shows a combination of measure and task. Points display the raw score per participant. Grey diamonds denote the group mean of each condition. See Table B4 for model output.

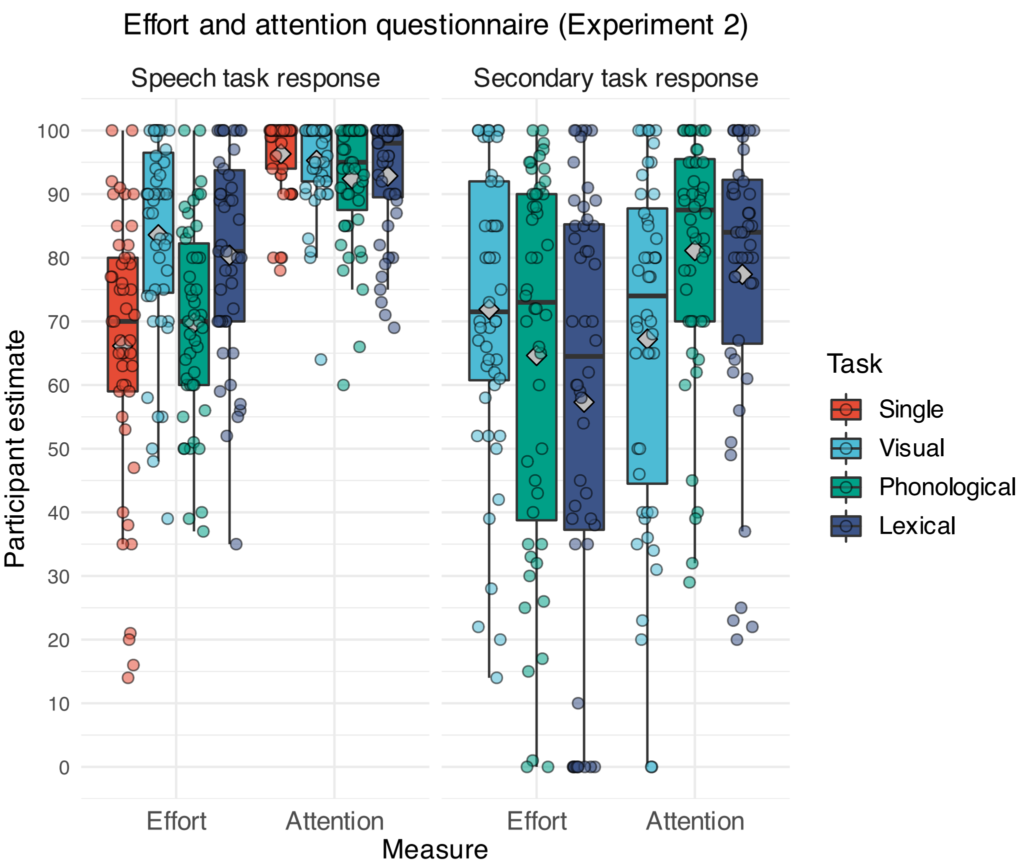

Figure C5. Participants' estimate on their effort and attention invested in the speech and secondary task under different task conditions in Experiment 2. The fill of the boxes represents task condition. Each panel shows a combination of measure and task. Points display the raw score per participant. Grey diamonds denote the group mean of each condition. See Table B7 for model output.

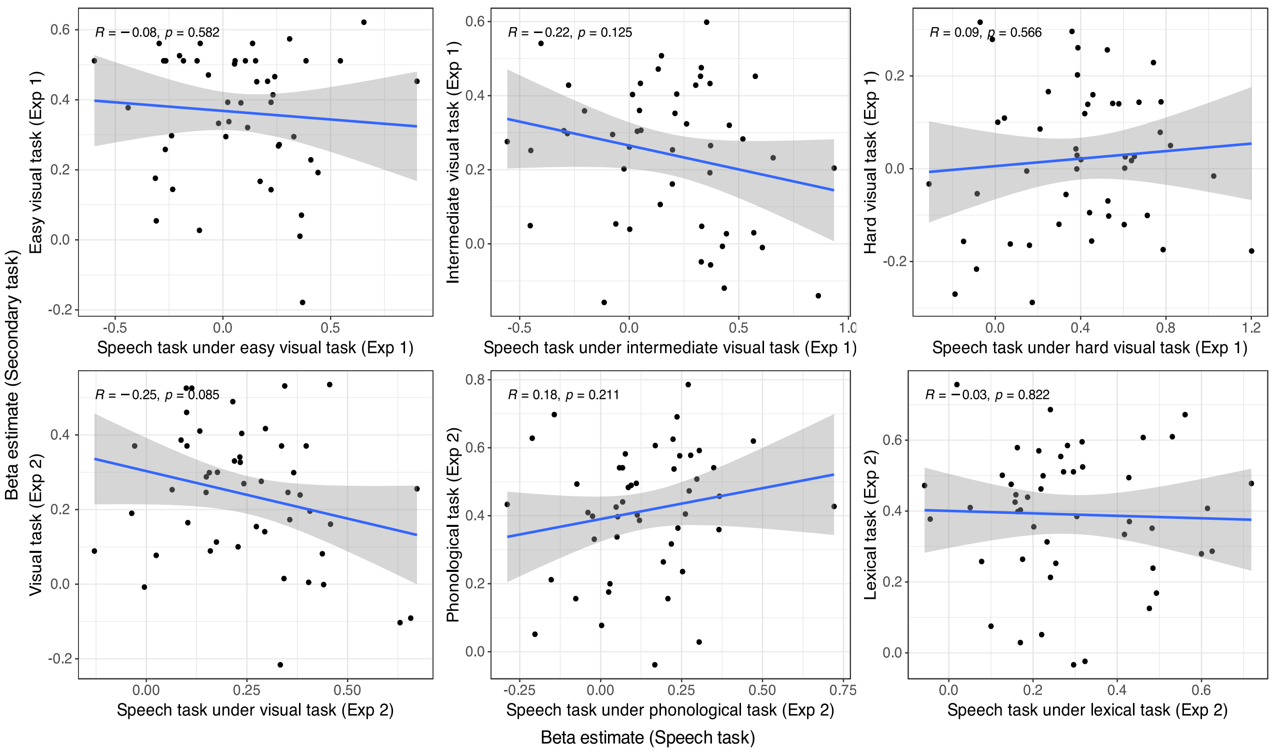

Figure C6. Beta estimates of the log(trial) term in the GLMMs for individual participants in different pairs of speech- and secondary-task conditions were extracted as a measure of individual slope of perceptual learning (i.e., a 1% increase in Trial leads to a change in the task performance for 1% of the value of the slope). Models were fitted for the speech % correct and secondary-task correctness in both experiment 1 and 2. All four models had Task, log(Trial), and their interaction as predictors, and only included performance for the first 20 trials where increased performance were observed for all task conditions. All models included random intercepts for Participant and random slopes for Trial by Participant. Each panel shows a combination of speech- and secondary-task conditions. Solid blue lines display the best-fitted linear functions from individual beta estimates in the speech-task conditions into their beta estimates in the secondary-task conditions. Points display the beta estimates per participant. Filled areas represent 95% confidence intervals. Pearson's correlation coefficient (R) and the corresponding *p* values are displayed at the top-left corner of each panel.

Appendix D

Effort and attention questionnaire (Experiments 1 and 2)

Those were all the 40 trials in the main task. Thank you for your participation so far! Before we send you onwards to Prolific, we have a few short questions which take around 5 minutes to complete. Please answer all of the questions, thanks!

Question 1: Please indicate using the slider below how **effortful** you found it to understand the **sentences**:

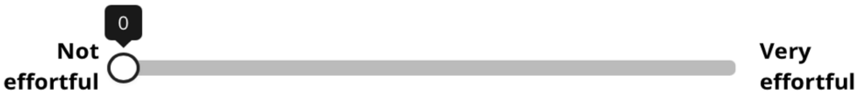

Question 2 (in all visual conditions, Experiment 1): Please indicate using the slider below how **effortful** you found it to decide the **angle of a patch**:

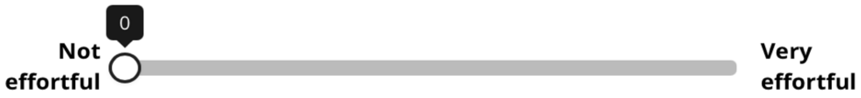

Question 2 (in visual condition, Experiment 2): Please indicate using the slider below how **effortful** you found it to decide the **angle of a word**:

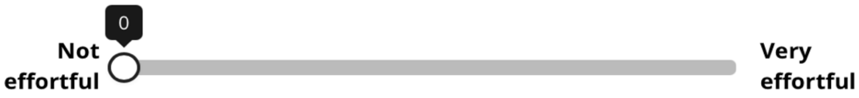

Question 2 (in phonological condition, Experiments 2): Please indicate using the slider below how **effortful** you found it to decide the **number of syllables in a word**:

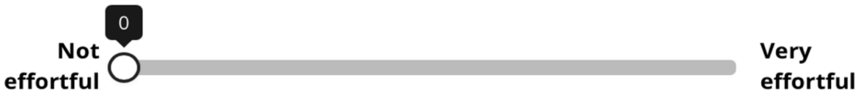

Question 2 (in lexical condition, Experiment 2): Please indicate using the slider below how **effortful** you found it to decide the **category of a word**:

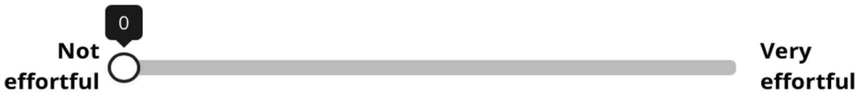

Question 3: Please indicate using the slider below how much **attention** you invested on understanding the **sentences**:

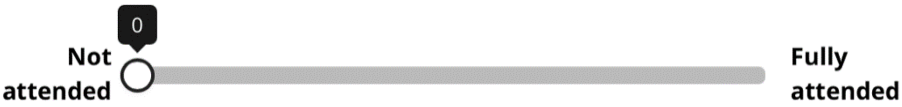

Question 4 (in all visual conditions, Experiment 1): Please indicate using the slider below how much attention you invested on deciding the **angle of a patch**:

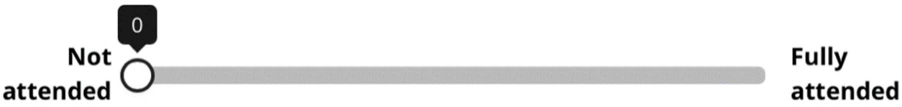

Question 4 (in visual condition, Experiment 2): Please indicate using the slider below how much attention you invested on deciding the **angle of a word**:

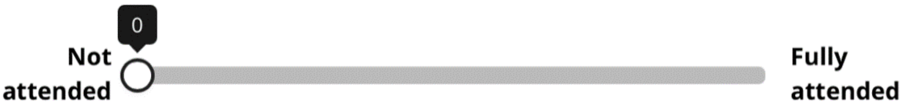

Question 4 (in phonological condition, Experiments 2): Please indicate using the slider below how much attention you invested on deciding the **number of syllables in a word**:

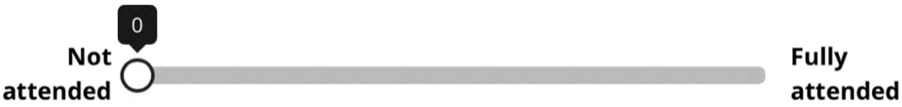

Question 4 (in lexical condition, Experiment 2): Please indicate using the slider below how much attention you invested on deciding the **category of a word**:

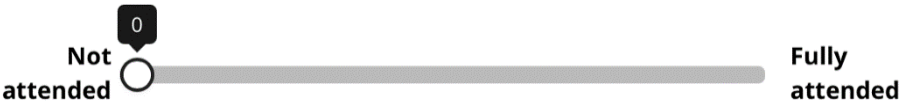

Appendix E

Supplementary data

The data files and R scripts used for conducting formal analyses and generating figures for both Experiments are available at: https://github.com/hwanguc/gam_glm_wang_etal_dual_task.

Credit author statement

Han Wang: Experimental design, data collection and analysis (Experiments 1 and 2), writing;

Rongru Chen and Yu Yan: Data collection and analysis (Experiment 2), writing;

Carolyn McGettigan and Stuart Rosen: Project supervision, writing;

Patti Adank: Experimental design, project supervision, writing.
